# Monkeys exhibit a paradoxical decrease in performance in high-stakes scenarios

**DOI:** 10.1073/pnas.2109643118

**Published:** 2021-08-23

**Authors:** Adam L. Smoulder, Nicholas P. Pavlovsky, Patrick J. Marino, Alan D. Degenhart, Nicole T. McClain, Aaron P. Batista, Steven M. Chase

**Affiliations:** ^a^Department of Biomedical Engineering, Carnegie Mellon University, Pittsburgh, PA 15213;; ^b^Center for the Neural Basis of Cognition, Carnegie Mellon University and University of Pittsburgh, Pittsburgh, PA 15213;; ^c^Department of Bioengineering, University of Pittsburgh, Pittsburgh, PA 15261;; ^d^Department of Electrical Engineering, Carnegie Mellon University, Pittsburgh, PA 15213;; ^e^Neuroscience Institute, Carnegie Mellon University, Pittsburgh, PA 15213

**Keywords:** motor control, reward, animal behavior, reaching, motivation

## Abstract

In high-stakes situations, people sometimes exhibit a frustrating phenomenon known as “choking under pressure.” Usually, we perform better when the potential payoff is larger. However, once potential rewards get too high, performance paradoxically decreases—we “choke.” Why do we choke under pressure? An animal model of choking would facilitate the investigation of its neural basis. However, it could be that choking is a uniquely human occurrence. To determine whether animals also choke, we trained three rhesus monkeys to perform a difficult reaching task in which they knew in advance the amount of reward to be given upon successful completion. Like humans, monkeys performed worse when potential rewards were exceptionally valuable. Failures that occurred at the highest level of reward were due to overly cautious reaching, in line with the psychological theory that explicit monitoring of behavior leads to choking. Our results demonstrate that choking under pressure is not unique to humans, and thus, its neural basis might be conserved across species.

## Significance

Choking under pressure is a frustrating phenomenon experienced sometimes by skilled performers as well as during everyday life. The phenomenon has been extensively studied in humans, but it has not been previously shown whether animals also choke under pressure. Here we report that rhesus monkeys also choke under pressure. This indicates that there may be shared neural mechanisms that underlie the behavior in both humans and monkeys. Introducing an animal model for choking under pressure allows for opportunities to study the neural causes of this paradoxical behavior.

In American football, situations occasionally arise where the outcome of a game or even an entire season comes down to a single action by a single player. During a 2015 National Football League (NFL) tournament game, the Minnesota Vikings faced a last-minute field goal attempt that, if successful, would have allowed them to win the game and advance in the playoffs. The placekicker had a 100% success rate for field goals at this distance throughout the regular season. This time, however, he missed the kick, costing the Vikings the game and ending their season. Failing like this when the stakes are high is no unique occurrence in the NFL: from 2001 to 2019, the success rate for regular season field goals at a distance between 40 and 55 yards from the goal posts was 75%. However, when less than two minutes of time remained in a game and a successful kick would tie or win, kicking accuracy fell to 66% (*SI Appendix*, *Materials and Methods*). Extensive analyses of American football and other sports have found that performance can suffer in high-stakes situations (1–4). This is the well-known effect of “choking under pressure”: performing worse than expected given your abilities (5–7).

Choking under pressure plagues ordinary people, not just professional athletes, and it has been demonstrated under a variety of controlled laboratory conditions, including challenging motor tasks (8, 9), test-taking (10), and performing in front of an audience (11, 12). The choking phenomenon is best represented by an “inverted-U” shape to the relationship between performance and incentive (13, 14). That is, performance improves with increasing reward, but only up to a point; for exceptionally high rewards, when it seems performance should matter the most, performance paradoxically declines.

Why do we choke under pressure? Numerous psychological explanations for choking have been offered, including social pressure (15, 16), loss aversion (17), distracting thoughts about possible rewards (7, 18, 19), over-arousal (20, 21), and explicit monitoring of performance (5, 8, 22, 23). Human imaging studies have identified differences in activity in several brain areas that correlate with choking propensity (17, 18). However, it has been difficult to link psychological explanations for choking to detailed neural mechanisms. This is in part because to date, no animal model for studying choking has been demonstrated. Knowing that animals also choke under pressure would permit researchers to probe the cellular-level mechanisms of the phenomenon. Also, showing that animals choke would suggest that this seemingly counterproductive behavior is evolutionarily conserved.

**Fig. 1.**
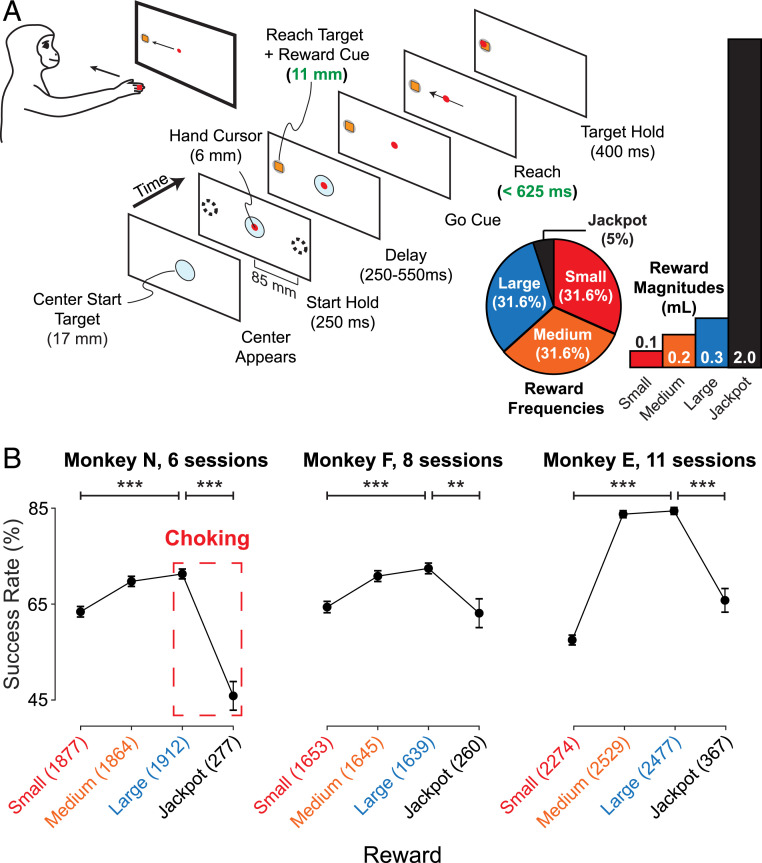
Monkeys choke under pressure. (*A*) Speed + accuracy task structure. Monkeys initiated trials by placing their hand so that a cursor (red circle) fell within the start target (pale blue circle). The reach target then appeared (gray circle with orange shape) at one of two (Monkeys N and F) or eight (Monkey E) potential locations (dashed circles), where the inscribed shape’s form (Monkey N) or color (Monkeys F and E) indicated the potential reward available for a successful reach. After a short, variable delay period, the start target vanished, cueing the animal to reach to the peripheral target. The animals had to quickly move the cursor into the reach target and hold for 400 ms before receiving the cued reward. Parameters for Monkey N are shown (*SI Appendix*, Table S1). Green font indicates parameters that were titrated to adjust the difficulty of the task. The frequency and magnitude of each reward cue is shown on the right. (*B*) All three animals showed a decrease in performance from the Large-to-Jackpot rewards—they “choked under pressure.” The points at each reward represent the average success rate across all trials for all sessions. Error bars represent the SE of the average success rate, calculated using a bootstrapping technique (*SI Appendix*, *SI Materials and Methods*). The number of trials presented for each reward cue are listed in parentheses next to the reward name. Significant differences in success rates between rewards were computed using a binomial proportion test. Stars indicate significance levels: **P < 0.01, ***P < 0.001. For visual clarity, we only show significant differences in performance between Small and Large rewards as well as Large and Jackpot. Note that for Monkey E, a variety of reward sizes were presented for the Small and Large reward cues (*SI Appendix*, *SI Materials and Methods*). Data shown here are for 0 and 0.4 mL; all data are shown in *SI Appendix*, Tables S1 and S2.

Here, we demonstrate that monkeys choke under pressure. We trained three rhesus monkeys to perform a challenging reaching task. The size of the potential reward was cued at the beginning of each trial. Reward sizes included the rare possibility of a “Jackpot” reward (Fig. 1*A*). We find that success rates on this task follow a classic inverted-U in which performance improved as reward sizes increased from Small to Large but then, paradoxically, declined for Jackpot rewards. Analysis of reaching kinematics indicates that monkeys choked under pressure in part because they reached too cautiously on Jackpot trials, a finding that is aligned with the “explicit monitoring” psychological account of choking (7, 22, 23).

## Results

To determine whether nonhuman animals choke under pressure, we trained three rhesus monkeys (*Macaca mulatta*) to perform a challenging delayed-reaching task that required them to plan and then execute reaches that are both fast and accurate (Fig. 1*A*), which we accordingly call the “speed + accuracy” task. We informed the animals of the potential rewards by presenting cues indicating which of four different reward sizes (Small, Medium, Large, or Jackpot) would be received upon successful completion of the task. The Jackpot reward was 10 times the size of the Medium reward and was presented on only 5% of trials. The animals understood the cues, as shown by a separate two-target choice task, in which they chose the target with the Jackpot reward 100% of the time, and they selected the target with the larger reward over 95% of the time overall (*SI Appendix*, Fig. S1 *A* and *B*). We titrated the difficulty of the task by reducing the target diameter and the time allowed to complete the reach (green font parameters in Fig. 1*A*). Experimental parameters for each subject are shown in *SI Appendix*, Table S1.

All three monkeys choked under pressure (Fig. 1*B*). Success rates improved from Small to Large rewards for each subject (N: 8.0%, F: 8.1%, and E: 24.5%; binomial proportion test, *P* < 10^−6^ for all), presumably because increasing reward size increased motivation. However, performance then paradoxically declined from Large to Jackpot rewards: Monkey N’s success rate fell by 25.2% (*P* < 10^−10^), Monkey F’s fell 9.6% (*P* = 1.5 × 10^−3^), and Monkey E exhibited a decrease of 17.2% (*P* < 10^−10^). *SI Appendix*, Table S2 provides a breakdown of trial counts, success rates, and statistical significance of differences between reward success rates for each individual session.

Humans choke across a range of behavioral paradigms (1, 2, 4, 5, 8–12). Is choking robust across tasks in monkeys as well? We trained one animal (Monkey F) to perform a “precision” task, in which the animal was instructed to follow a path from the center of the screen to the reach target (*SI Appendix*, Fig. S2A and *SI Materials and Methods*) (24). While movements were not required to be fast like those of the original task, the cursor had to remain within the visible boundary. We collected 29 sessions of precision task data from this monkey, which provided a total of 611 Jackpot reward trials. The animal also choked under pressure in this task (*SI Appendix*, Fig. S2*B*, black line).

Even with years of practice and experience under pressure, professional athletes can still choke (1–4), implying that choking cannot be overcome through extensive practice. Similarly, in the speed + accuracy task, we observed that choking occurred across many sessions, suggesting monkeys also could not easily overcome the behavior. Monkey N choked under pressure in 6/6 sessions, Monkey F in 7/8 sessions, and Monkey E in 11/11 sessions (*SI Appendix*, Fig. S3*A*). We also note that after nearly a month of exposure to tasks with a Jackpot reward, Monkey F still showed significant choking under pressure (*SI Appendix*, Fig. S2*B*, olive line). Further, choking occurred in both the first and second half of the individual experiment day (*SI Appendix*, Fig. S3*B*), suggesting that the effect was relatively robust to the state of satiation and instead may be driven by a stable understanding of the Jackpot reward’s relative value. The persistence of choking across and within sessions leads us to conclude that choking under pressure in monkeys is a robust and reliable effect, consistent with the phenomenon in humans.

### Both Jackpot Reward Rarity and Magnitude Were Needed to Induce Choking.

In humans, choking tends to occur in situations of unusually high stakes. However, what defines “high stakes”? In our paradigm, the Jackpot reward that caused choking was both rarer than rewards of other sizes and also greater in magnitude. Can either rarity or magnitude alone account for the decrease in performance we observed, or are both necessary to cause choking? We performed two additional experiments in one animal (E) to separately examine the behavioral effects of the Jackpot’s rarity and magnitude.

**Fig. 2.**
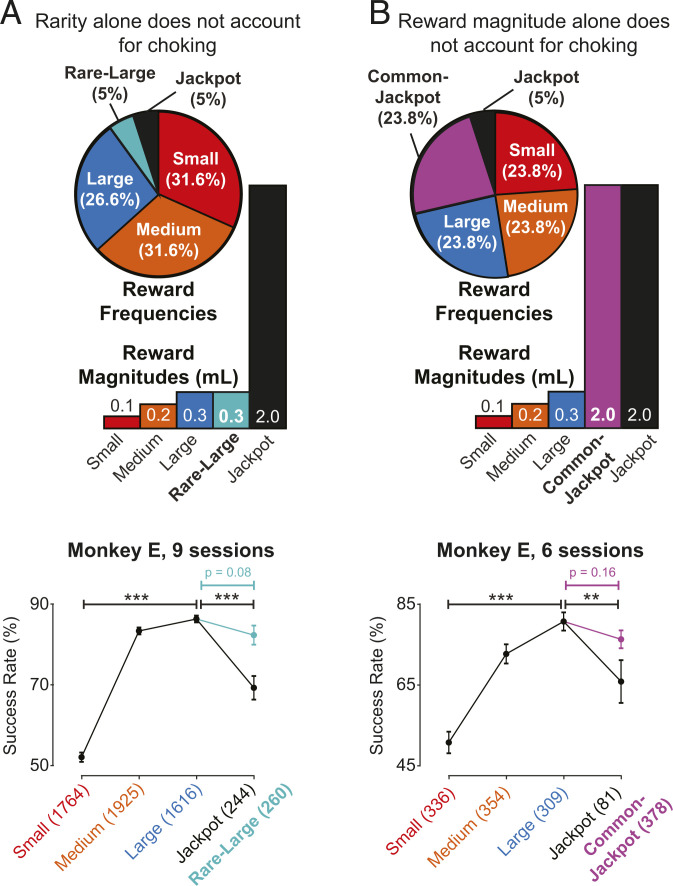
Neither rarity nor reward magnitude alone are sufficient to induce choking. Monkey E performed additional experiments of the speed + accuracy task with a fifth reward cue. (*A*) A “Rare-Large” (cyan) reward cue matched Large rewards in magnitude but Jackpot rewards in frequency (*Top*). Success rates for each reward are shown (*Bottom*). The cyan data point shows the Rare-Large success rate, which was not significantly lower than that of Large rewards. Black data points and significance bars indicate the performance values recorded on these sessions with the standard four rewards. Stars indicate significance levels: **P < 0.01, ***P < 0.001. (*B*) A “Common-Jackpot” (purple) reward cue matched the Jackpot reward magnitude but the frequency of non-Jackpot rewards (*Top*). Performance and significant differences in success rates across rewards are shown (*Bottom*), in which purple bars show the relationship between Large and Common-Jackpot success rates. For both experiments, we ensured that Monkey E understood the relative value of the new reward cues using a two-target choice task (*SI Appendix*, Fig. S1 *C* and *D*).

To test whether rarity was sufficient to induce choking, we ran a control set of experiments in which we introduced a fifth reward cue into the speed + accuracy task. This “Rare-Large” cue was as rare as the Jackpot reward, but it signaled a reward of the same magnitude as the Large reward (Fig. 2 *A*, *Top*). If rarity alone can cause choking, we would expect that performance for the Rare-Large and Jackpot rewards would be commensurate. This was not the case (Fig. 2*A*, *Bottom*): while the animal still choked for the Jackpot reward (14.3% decrease from Large, *P* = 7.9 × 10^−9^), there was only a small, nonsignificant performance decrement from Large to Rare-Large (4.1% decrease, *P* = 0.075), and the success rate for the Rare-Large trials was significantly higher than for the Jackpot (*SI Appendix*, Table S2, 10.2% difference, *P* = 6.4 × 10^−3^). This result is supported by observations we made during pilot sessions of the precision task in Monkey F. In these sessions, a smaller, “Mini-Jackpot” reward of 0.8 mL was used, and the animal exhibited a small, nonsignificant performance decrement between the Large and Mini-Jackpot rewards (*SI Appendix*, Fig. S2*C*, 2.7% drop, *P* = 0.24). Together, these results indicate that a cue’s rarity on its own has only a small effect on performance.

To determine whether the Jackpot’s magnitude was sufficient to cause choking, we ran a different control set of experiments in which we again introduced a fifth reward cue into Monkey E’s speed + accuracy task. This “Common-Jackpot” cue had the same magnitude as the Jackpot reward, but it appeared just as frequently as did the non-Jackpot rewards (Fig. 2 *B*, *Top*). If reward magnitude alone accounted for choking, we would expect commensurate performance between the Common-Jackpot and Jackpot rewards. However, this is not what we observed (Fig. 2 *B*, *Bottom*): again, the animal choked for the Jackpot reward in these sessions (14.8% decrease from Large, *P* = 0.0059), but the performance decrement from Large to Common-Jackpot was small and nonsignificant (4.5%, *P* = 0.16). Combined, the results from these two control experiments indicate that both the Jackpot reward’s rarity and magnitude are needed to induce the animals to choke under pressure in our task.

### Monkeys Choked Due to Approaching the Target with Excess Caution.

What behavioral changes led to choking under pressure? Here, we report on two trends in behavior that exacerbate as reward size increases. Then, we show how these behaviors lead to choking in our speed + accuracy task.

**Fig. 3.**
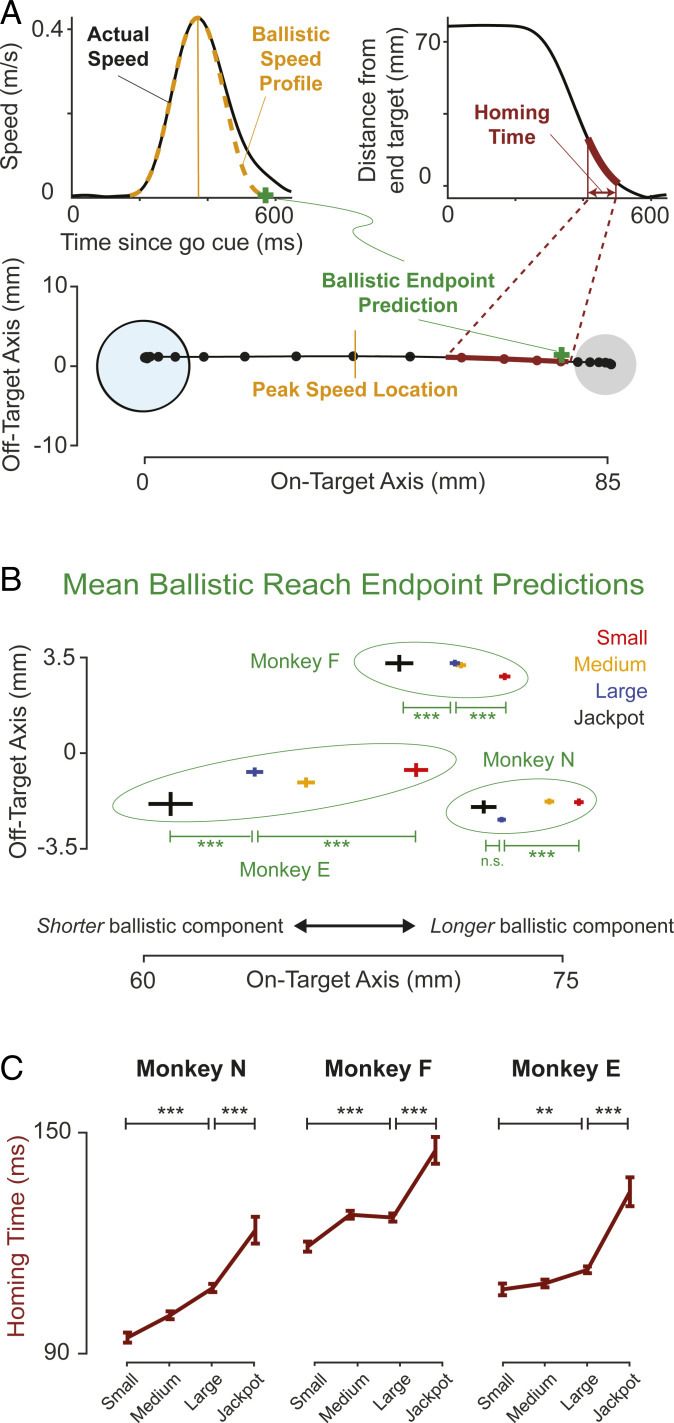
Animals reach more cautiously with increasing rewards. (*A*) Illustration of behavioral analysis techniques. (*Left*) Calculation of ballistic reach endpoint. For each trial, the velocity to peak speed was mirrored across time, and the endpoint of this new, mirrored trace is the “ballistic endpoint prediction.” (*Right*) “Homing time” is how long it took the animal to move from 2/3 the distance to the target to within 1 mm. (*Bottom*) An example reach trajectory with dots every 25 ms and with both the ballistic endpoint prediction and homing time period shown. (*B*) Mean ballistic reach endpoint predictions as a function of reward (error bars represent SE). Stars indicate significance levels: **P < 0.01, ***P < 0.001. *P* > 0.05 for comparisons listed as not significant (n.s.). (*C*) Median homing times versus reward (error bars are SE of median; *SI Appendix*, *SI Materials and Methods*). Homing time trends were robust to changes in the distance over which it was calculated (i.e., last 1/2 or 1/4 of the distance; *SI Appendix*, *SI Materials and Methods*).

Reaches can be decomposed into two phases: an initial accelerating phase and a subsequent decelerating phase. The initial phase is probably unaffected by visual feedback, and the decelerating phase probably involves adjusting the movement to “home in” on the target. Thus, we refer to the initial portion of the reach as the “ballistic” component and the final portion as the “homing” component (25–28). To determine the behavioral changes that led to the animals choking under pressure, we first examined the “ballistic” portion of the reach. We estimated where the reach would have landed if there had been no error correction under visual feedback. To do this, we mirrored the velocity profile from the start of the reach until peak speed, then integrated it across time, resulting in a “ballistic reach endpoint prediction” (Fig. 3 *A*, *Top Left* and *Bottom*; *SI Appendix*, *SI Materials and Methods*). We examined these ballistic reach endpoint predictions and found that they became shorter with higher reward sizes (Fig. 3*B*), implying that as the reward size increased, less of the distance to the target was covered by the initial portion of the reach.

We then considered the “homing” component of the reach. We examined how long the animals spent approaching the target and how that time depended on reward. We calculated the “homing time” for each trial as the amount of time that it took for the animal to move the final third of the distance to the target (Fig. 3 *A*, *Top Right* and *Bottom*, and *SI Appendix*, *SI Materials and Methods*). Homing time increased progressively with reward (Fig. 3*C*), indicating that the animals spent a greater amount of time homing in on the target when rewards were higher. The homing time trends were not solely the result of overall slower reaching, as we discuss in *SI Appendix*, Fig. S4 and in *Additional Idiosyncratic Factors Also Contribute to Choking*. We note that these behavioral changes may be rational when optimizing reaches for greater accuracy (29). Together, these behavioral trends are consistent with the animals exhibiting increasing caution as reward sizes increase: they attain their peak velocities further from the reach target and spend more time homing in to approach the target.

**Fig. 4.**
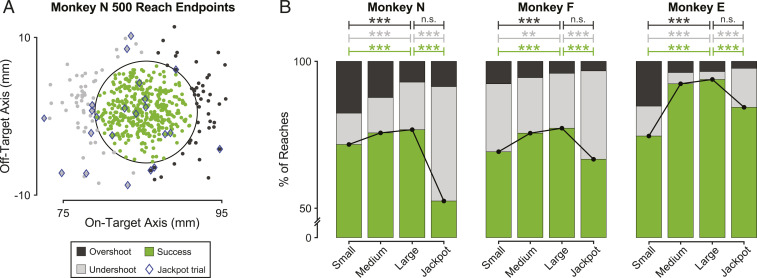
The tradeoff between undershoots and overshoots depends on reward size in a manner that leads to choking under pressure. (*A*) Example reach endpoints from one subject to the rightward target (Monkey N; *SI Appendix*, *SI Materials and Methods*). Each trial was an accurate reach (green) or a failure due to undershoot (light gray) or overshoot (dark gray). Jackpot trials are highlighted with blue diamonds. Note these are actual reach endpoints and not the predicted ballistic endpoints examined in Fig. 3*B*. The black circle indicates the size and location of the reach target. (*B*) Incidence of different reach outcomes shown as a function of reward. The black line connects the rates of successful reaches to highlight the “inverted-U” effect of reward on success rate. Stars indicate significance levels: **P < 0.01, ***P < 0.001. *P* > 0.05 for comparisons listed as not significant (n.s.). *P* values for all comparisons are listed in *SI Appendix*, Tables S3 and S4.

How might these trends in reaching behavior that are monotonic with reward magnitude lead to the inverted-U characteristic of choking under pressure? To answer this question, we must consider how these kinematic metrics relate to performance. By the design of the speed + accuracy task, there are three ways that a reach to the target can end. First, the animal could successfully stop its hand in the target (Fig. 4*A*, green). The second possibility is that the animal does not reach far enough, either landing short of the target or being midreach when time expires (“undershoot” failures, Fig. 4*A*, light gray). Third, the animal could reach through or past the target without stopping in it (“overshoot” failures, Fig. 4*A*, dark gray). If the animal approaches the target too cautiously, they will exhibit more undershoot failures. Conversely, overshoots may indicate less careful reaching; for example, stemming from a longer ballistic component of the movement.

The fraction of total reaches that fell into each of these three categories depended on reward magnitude (Fig. 4*B*). For all three animals, the proportion of undershoot failures increased from Large to Jackpot. This is the manifestation of the overcaution shown in Fig. 3. Also, in comparing Small to Large rewards, the proportion of overshoot failures was greater for Small reward trials, perhaps indicating a lack of regard for precision when the potential payoff was low. The combination of more frequent overshoot errors for Small rewards and undershoot errors for Jackpots are sufficient to create the inverted-U relationship between success rate and reward size that characterizes choking under pressure (*SI Appendix*, *SI Materials and Methods*).

These consistent trends with reward size suggest that there is a “sweet spot” along the reward continuum, with optimal performance occurring for Large rewards. On Small reward reaches, the animals appear to reach carelessly, overshooting more often, and they do not seem motivated to perform these movements as reliably as they are capable of. In contrast, on Jackpot reward reaches, animals come up short, seemingly because they are being overly cautious. Our results indicate that all animals choked under pressure at least in part due to an apparent excess of caution in approaching the target for Jackpot rewards.

### Additional Idiosyncratic Factors Also Contribute to Choking.

All three animals reached more cautiously as the potential reward got larger, but this was not the sole source of choking behavior. In humans, choking manifests differently across individuals in both manner and extent (14). Similarly, the animals in our experiments exhibited idiosyncratic behavioral changes that also resulted in choking. We considered separately each epoch of the speed + accuracy task: delay, reach, and target-hold. In each epoch, we found behavioral and performance trends that differed across animals to produce unique choking behavior in each. As described above, choking behavior during the reach epoch, interpretable as overcaution, was evident in all animals. Below, we also discuss the other two epochs of the tasks, in which trends were not consistent across all three monkeys.

We first examined behavior during the delay epoch, encompassing the time after target onset but before the go cue (Fig. 1*A*). Trial failure during the delay epoch could occur if animals commenced a reach before the go cue (false starts, *SI Appendix*, Fig. S5 *A*, *Top Left*) or if they drifted out of the center start target before the go cue (delay drifts, *SI Appendix*, Fig. S5 *A*, *Middle*). All monkeys exhibited the inverted-U in success rates that indicates choking in the delay epoch, and when false starts and delay drifts were examined separately, each showed the inverted-U relationship on its own. These effects were not statistically significant for Monkey F but were particularly substantial for Monkey E. Full results are listed in *SI Appendix*, Tables S3 and S4. It is hard to interpret starting too soon or drifting out of the start target as overcaution. Instead, this behavior might be better described as impulsivity (13, 20, 21), which suggests that diverse behavioral mechanisms might be at play when choking occurs. This is consistent with the variety of explanations offered in the human behavioral literature on choking (7, 19–21).

If the decrease in delay epoch performance for Jackpot trials indicated increased impulsivity, we might expect to see faster reaction times and peak speeds in the reach epoch (*SI Appendix*, Fig. S5 *B*, *Left*). This was not consistently the case (*SI Appendix*, Fig. S5 *B*, *Right*). Instead, reaction time and peak speed showed idiosyncratic changes with reward for each subject, again supporting the possibility of a diversity of separate behavioral explanations for choking.

Lastly, we consider the target-hold epoch. Even if the subject successfully lands within the reach target, they still have to hold the cursor within the target for 400 ms for the trial to be successful. If the hand drifts out of the end target (“target hold drift”), they fail (*SI Appendix*, Fig. S5 *C*, *Left*). This behavioral tendency could be interpreted as a manifestation of “nerves.” We looked at successes versus target hold drifts out of all of the trials that landed within the reach target (*SI Appendix*, Fig. S5 *C*, *Right*). Monkey N exhibited significant choking due to target hold drifts, but other monkeys did not. For completeness, all analyses discussed here were also performed on the Rare-Large and Common-Jackpot data and are presented in *SI Appendix*, Fig. S6.

To summarize these trends, a tendency to choke (that is, to underperform for a Jackpot reward) was observed in every epoch of the task for at least one monkey, but only during the reach epoch was consistent, significant choking behavior seen in all three animals. It is possible that these diverse manifestations of choking behavior may all share a common underlying explanation in terms of emotional states triggered when the stakes are high (5, 7, 30).

## Discussion

Three monkeys performed a challenging reaching task in which they knew in advance the magnitude of the reward they would earn for a successful movement. We found that monkeys choke under pressure, just as humans do. This effect is robust in that it does not go away with extended practice; it persists despite changes in satiation, and it occurs in challenging tasks with very different motor demands. All of our animals responded to increases in reward by shortening the initial component of their reach and then taking longer to home in on the target, ultimately leading to choking via an excess of short reach (undershoot) failures at the highest level of reward. Conversely, all animals were more prone to overshoot the target when smaller rewards were proffered. This and other aspects of behavior show that there is a marked difference in kinematics for Small-reward and Jackpot-reward trials, even though failures are of comparable prevalence. We also observed a number of idiosyncratic changes in behavior with reward, reminiscent of the variety of responses that humans exhibit in the face of pressure (14, 31).

### Choking Occurs When Rare and Exceptionally Large Rewards Are Proffered.

To induce choking under pressure, the magnitude of the reward had to be high and its probability of occurring had to be low. Magnitude alone and rarity alone were not sufficient to elicit the large choking effect we observed (Fig. 2 and *SI Appendix*, Fig. S2*C*). These results also rule out the possibility that the observed choking is solely due to surprise: we did not see a significant performance decrement for Rare-Large rewards, even though they occurred as infrequently as Jackpot rewards.

In sessions during which we presented both Common-Jackpot cues and rare-Jackpot cues, the animal choked for rare-Jackpot trials but not Common-Jackpot trials (Fig. 2*B*). We can think of two potential explanations for this intriguing behavior. First, it may be that the rare-Jackpot reward was actually of higher perceived value than the Common-Jackpot reward. In support of this view, in the two-target choice task, the monkey chose the rare-Jackpot target rather than the Common-Jackpot target in four of five trials (*SI Appendix*, Fig. S1*D*; note that only five trials of this comparison were run, because we did not want to disrupt the animal's understanding of the frequencies of these cues). This is perhaps not surprising, as novelty itself can be intrinsically rewarding (32, 33). A second possibility is that the animal had a conditioned response to the rare-Jackpot cue after extended exposure; that is, he was so used to a particular behavioral response to that cue that he never changed it, even when the same magnitude of reward was associated with multiple cues. If true, this has the interesting implication that choking under pressure might actually become a learned phenomenon, like a bad habit. In further support of this possibility is our observation that the choking behavior was not eliminated with satiety (*SI Appendix*, Fig. S3*B*). This perspective might be valuable when considering the design of training regiments that could help professional athletes and others become less prone to choking under pressure.

### Links to Psychological Theories of Choking.

Here, we adopt the operational definition of choking offered from Baumeister (5), as “performance decrements under circumstances that increase the importance of good or improved performance.” This seminal definition is objective and behavioral, and as such, it leaves a psychological explanation for choking under-determined, and it also does not address the ultimate neurobiological source of choking. We conducted a close analysis of our animals' kinematic data to see whether we could find support for one or more of the psychological-level explanations for choking.

Psychological theories of choking fall predominantly into three categories: explicit monitoring, over-arousal, and distraction (8, 10, 14, 18). Explicit monitoring posits that choking occurs when normally autonomous actions receive active scrutiny, which can lead to failure via slowed movements, poorer timing, or worse coordination (22, 23). Over-arousal theories posit that high stakes or rewards drive the internal state of arousal beyond an optimal range, leading to poor performance (17, 20, 21). Meanwhile, distraction theories posit that the source of pressure takes up valuable working memory and attentional resources that might otherwise have been devoted to the task (7, 10, 18, 19). Of course, these explanations are not mutually exclusive, and in fact, all of these processes may interact.

To our knowledge, this type of skilled reaching task has not been used previously to study choking under pressure in the laboratory. It is not straightforward to make a one-to-one correspondence between the psychological theories of choking and the changes in kinematics that they might predict. Nevertheless, we can argue in support of some explanations based on the behavioral manifestations that we observe. We see our findings as most consistent with an explicit monitoring explanation for choking under pressure. One way to interpret explicit monitoring is that it makes one over-reliant on sensory feedback. The shortening of the initial component of the reach and the increase in homing time with increases in reward are consistent with this. These changes may indicate a desire on the part of the animal to prioritize accuracy over speed, since accuracy is the more-evident indicator of success in the speed + accuracy task. All three animals exhibited this behavior, and thus, we posit that explicit monitoring seems to be the dominant factor underlying choking in our task. However, it was not the only factor. For example, we also saw behavioral changes that we can interpret as over-arousal (34, 35). Monkey F showed a decrease in reaction time with increasing rewards (*SI Appendix*, Fig. S5*B*), and all monkeys showed an increased incidence of false starts from the Large to Jackpot rewards (*SI Appendix*, Fig. S5*A*). Monkeys N and E showed increases in reaction time from Large to Jackpot rewards, which is arguably consistent with distraction theories of choking, although since the animals sat alone in a darkened room, any source of distraction must be purely internal.

These psychological explanations for choking can provide some signposts toward identifying the neurobiological source of choking under pressure. We join others in speculating that the ultimate driver of choking is internal emotional states, such as anxiety, reward anticipation, arousal, or a combination of these (30, 36). Thus, choking might be tightly tied to neuromodulatory systems in the brain (6, 37, 38). This intriguing prospect heightens the value of this first demonstration of choking in a nonhuman animal, since in the future, invasive neural recordings might reveal the neuromodulatory drivers of choking under pressure. We conclude by considering how emotional states might interact with motor control signals.

### Relationships to Theories of the Neural Basis of Choking Under Pressure.

What might be the neural basis of choking under pressure? Skilled motor behavior presumably requires precise neural activity patterns to be formed in the cerebral cortex (39, 40). Reward signals have been reported throughout the cerebral cortex (41–44), including in areas involved in motor planning (41, 45) and in motor cortex (42, 46–48). It may be that reward anticipation signals help boost the quality of cortical motor signals, but only up to a point; when these signals become unusually large, they may actually degrade the quality of movement-related signals. For example, dopamine neuron activity is known to scale with the magnitude of an anticipated reward (49, 50). The inverted-U relationship present in the animals' success rates resembles those seen in studies relating the activity of neuromodulators such as dopamine and norepinephrine to performance in working memory, visuomotor, and attention tasks (37, 38, 51, 52). These studies demonstrate that a graded release of a neurotransmitter can lead to discontinuities in behavioral performance. An appealing aspect of a neuromodulatory explanation of choking is that the same mechanism can explain why choking occurs in motor tasks (as shown here) as well as in cognitive tasks (10). There is, of course, not necessarily a solitary neural mechanism for choking under pressure, a view which is supported by the fact that different psychological explanations have been proposed and different brain areas have been implicated in choking (8, 17, 18). However, the same underlying “cause” of choking behavior, such as the activity of a neuromodulator like dopamine, may have different impacts on different recipient brain areas. Because different neurophysiological pathways are implicated in each of the psychological theories (17, 18), invasive neural recordings may provide an avenue to characterize this mixture of causes at a high spatial and temporal resolution, on a subject-by-subject and task-by-task basis. Given that we now know that both monkeys and humans choke under pressure, it is reasonable to assume that at least some of the neural mechanisms that lead to choking are conserved between them. Thus, this study elevates the prospect of identifying the neural mechanisms of choking, because an animal model for this curious phenomenon now exists.

## Materials and Methods

### Subjects.

Three adult male rhesus monkeys (*M. mulatta*, Monkeys N, F, and E) were used in this study. All animal procedures were approved by the University of Pittsburgh Institutional Animal Care and Use Committee and were in accordance with the guidelines of the US Department of Agriculture, the International Association for the Assessment and Accreditation of Laboratory Animal Care, and the NIH.

### Tasks.

Equipment information and details for all tasks are provided in *SI Appendix*, *SI Materials and Methods*. Parameters for each task layout and reward distribution are listed in *SI Appendix*, Table S1.

#### Speed + accuracy task.

The speed + accuracy task design is shown in Fig. 1*A*. The animals acquired a center target with a hand-controlled cursor to begin a trial. After 200 ms, a peripheral reach target appeared in one of two (Monkeys N and F) or eight (Monkey E) locations. Inside the target, an inscribed shape’s form (Monkey N) or color (Monkeys F and E) indicated the reward size that would be received upon trial success. Reward size was drawn independently for each trial. After holding the cursor at the center for a variable delay period, the center target disappeared as a go cue. The animals had a limited time to reach to the end target. They then had to hold the cursor within it for 400 ms before reward was administered.

We identified a total of five primary ways that the animals could fail a trial in this task (Fig. 3*A* and *SI Appendix*, Fig. S5 *A* and *C**)*, referred to as false starts, delay drifts, overshoots, undershoots, and target hold drifts. These, along with any other ways that a trial could be failed, are defined in detail in *SI Appendix*, *SI Materials and Methods*. Each failed trial was given one categorical label.

#### Two-target choice task.

We tested that the animals understood the relative value of the reward cues using a two-target choice task (*SI Appendix*, Fig. S1). The task was similar to the speed + accuracy task, with the key difference being the simultaneous presentation of two diametrically opposed reaching targets. We presented trials with differing target reward values to assess the animals’ valuation of the cues and also showed cases with the same reward values to assess any bias in the direction in which the animals preferred to reach.

#### Precision task.

Monkey F performed 29 sessions of a precision reaching task designed to assess whether choking would occur in conditions that did not necessitate fast movements (*SI Appendix*, Fig. S2). The general design was the same as the speed + accuracy task, except that the subject had to keep the cursor along a preset path from the center to the reach target and had a lax (2,000 ms) reach time constraint (24). Earlier, we performed 28 sessions of this task with a smaller Jackpot reward size.

### Data Analysis.

Due to the low numbers of Jackpot trials within individual sessions, we evaluated our data by pooling trials across all sessions (with the exceptions of *SI Appendix*, Fig. S3*A* and Table S2). Details for all analyses are provided in *SI Appendix*, *SI Materials and Methods*.

#### Statistics and error bars.

For comparisons across reward sizes involving rates of an event (i.e., success rates or rates of specific failure modes), we used a two-tailed binomial proportion test comparing Small-to-Large and Large-to-Jackpot conditions. For comparisons across reward sizes involving peak speed and ballistic endpoint predictions, we used Welch's *t* test for unequal sample sizes. We compared reaction time and homing time across reward sizes using the median to prevent the heavy tails of the distribution from affecting interpretation, and we used a Mann–Whitney *U* test to evaluate significant differences. For all of these summary metrics (success rates, medians, and means), we calculated SE bars with a bootstrapping procedure. To do this, within each subject and reward, we resampled (with replacement) a new set of trials equal to the original number from the data and calculated the metric of interest. We did 10,000 of these bootstraps and calculated the SE of the metric as the SD of these bootstraps.

#### Kinematic analysis.

Fig. 3 shows two key kinematic metrics calculated from the speed + accuracy task data for each subject and reward size. To calculate ballistic endpoint predictions, we first found the time of peak speed. We then took the velocity profile from the go cue until this time and reflected it around the time of peak speed. The resulting symmetric velocity profile is then integrated to find the displacement from the position of go cue. For homing time, we wanted a metric that reflected time spent near or approaching the reach target. To calculate this, we used the time it took the animals to cover the distance starting when the cursor was 2/3 of the way to the target and ending when the cursor first reached within 1 mm of it. The latest point in the trial at which homing time could be completed was set to be 150 ms after the reach failure timeout. We selected this 1-mm and 150-ms buffer to permit undershoot failures inclusion, in which the subject’s trajectory often had them very close to achieving the reach target near the time of failure. Altering these parameters did not change interpretation of the results (*SI Appendix*, *SI Materials and Methods*).

## Supplementary Material

Supplementary File

## Data Availability

.mat files of kinematic data have been deposited in Figshare (DOI: 10.6084/m9.figshare.13547435) (53). All code used for analysis is available in GitHub at https://github.com/adam-smoulder/MonkeysChokeUnderPressure_behavior.

## References

[1] N.-W. Hsu, K.-S. Liu, S.-C. Chang, Choking under the pressure of competition: A complete statistical investigation of pressure kicks in the NFL, 2000–2017. PLoS One 14, e0214096 (2019). 30939137 10.1371/journal.pone.0214096 PMC6445473

[2] A. M. Fryer, G. Tenenbaum, G. M. Chow, Linking performance decline to choking: Players’ perceptions in basketball. J. Sports Sci. 36, 256–265 (2018). 28271958 10.1080/02640414.2017.1298829

[3] N. Goldschmied, M. Nankin, G. Cafri, Pressure kicks in the NFL: An archival exploration into the deployment of time-outs and other environmental correlates. Sport Psychol. 24, 300–312 (2010).

[4] D. F. Gucciardi, J.-L. Longbottom, B. Jackson, J. A. Dimmock, Experienced golfers’ perspectives on choking under pressure. J. Sport Exerc. Psychol. 32, 61–83 (2010). 20167952 10.1123/jsep.32.1.61

[5] R. F. Baumeister, Choking under pressure: Self-consciousness and paradoxical effects of incentives on skillful performance. J. Pers. Soc. Psychol. 46, 610–620 (1984). 6707866 10.1037//0022-3514.46.3.610

[6] R. Yu, Choking under pressure: The neuropsychological mechanisms of incentive-induced performance decrements. Front. Behav. Neurosci. 9, 19 (2015). 25713517 10.3389/fnbeh.2015.00019 PMC4322702

[7] C. Mesagno, J. Beckmann, Choking under pressure: Theoretical models and interventions. Curr. Opin. Psychol. 16, 170–175 (2017). 28813345 10.1016/j.copsyc.2017.05.015

[8] S. L. Beilock, T. H. Carr, On the fragility of skilled performance: What governs choking under pressure? J. Exp. Psychol. Gen. 130, 701–725 (2001). 11757876

[9] R. C. Jackson, K. J. Ashford, G. Norsworthy, Attentional focus, dispositional reinvestment, and skilled motor performance under pressure. J. Sport Exerc. Psychol. 28, 49–68 (2006).

[10] S. L. Beilock, T. H. Carr, When high-powered people fail: Working memory and “choking under pressure” in math. Psychol. Sci. 16, 101–105 (2005). 15686575 10.1111/j.0956-7976.2005.00789.x

[11] C. Mesagno, J. T. Harvey, C. M. Janelle, Choking under pressure: The role of fear of negative evaluation. Psychol. Sport Exerc. 13, 60–68 (2012).

[12] H. M. Wallace, R. F. Baumeister, K. D. Vohs, Audience support and choking under pressure: A home disadvantage? J. Sports Sci. 23, 429–438 (2005). 16089187 10.1080/02640410400021666

[13] R. M. Yerkes, J. D. Dodson, The relation of strength of stimulus to rapidity of habit-formation. J. Comp. Neurol. Psychol. 18, 459–482 (1908).

[14] R. F. Baumeister, C. J. Showers, A review of paradoxical performance effects: Choking under pressure in sports and mental tests. Eur. J. Soc. Psychol. 16, 361–383 (1986).

[15] C. E. Kimble, J. S. Rezabek, Playing games before an audience: Social facilitation or choking. Soc. Behav. Personal. 20, 115–120 (1992).

[16] J. L. Butler, R. F. Baumeister, The trouble with friendly faces: Skilled performance with a supportive audience. J. Pers. Soc. Psychol. 75, 1213–1230 (1998). 9866184 10.1037//0022-3514.75.5.1213

[17] V. S. Chib, B. De Martino, S. Shimojo, J. P. O’Doherty, Neural mechanisms underlying paradoxical performance for monetary incentives are driven by loss aversion. Neuron 74, 582–594 (2012). 22578508 10.1016/j.neuron.2012.02.038 PMC3437564

[18] T. G. Lee, S. T. Grafton, Out of control: Diminished prefrontal activity coincides with impaired motor performance due to choking under pressure. Neuroimage 105, 145–155 (2015). 25449744 10.1016/j.neuroimage.2014.10.058 PMC4262628

[19] J. Wine, Test anxiety and direction of attention. Psychol. Bull. 76, 92–104 (1971). 4937878 10.1037/h0031332

[20] D. Ariely, U. Gneezy, G. Loewenstein, N. Mazar, Large stakes and big mistakes. Rev. Econ. Stud. 76, 451–469 (2009).

[21] J. A. Easterbrook, The effect of emotion on cue utilization and the organization of behavior. Psychol. Rev. 66, 183–201 (1959). 13658305 10.1037/h0047707

[22] R. S. W. Masters, Knowledge, knerves and know‐how: The role of explicit versus implicit knowledge in the breakdown of a complex motor skill under pressure. Br. J. Psychol. 83, 343–358 (1992).

[23] B. P. Lewis, D. E. Linder, Thinking about choking? Attentional processes and paradoxical performance. Pers. Soc. Psychol. Bull. 23, 937–944 (1997). 29506446 10.1177/0146167297239003

[24] P. T. Sadtler, S. I. Ryu, E. C. Tyler-Kabara, B. M. Yu, A. P. Batista, Brain-computer interface control along instructed paths. J. Neural Eng. 12, 016015 (2015). 25605498 10.1088/1741-2560/12/1/016015 PMC4330471

[25] R. S. Woodworth, Accuracy of voluntary movement. Psychol. Rev. 3, i-114 (1899).

[26] W. D. A. Beggs, C. I. Howarth, The accuracy of aiming at a target. Some further evidence for a theory of intermittent control. Acta Psychol. (Amst.) 36, 171–177 (1972). 5073207 10.1016/0001-6918(72)90001-7

[27] D. E. Meyer, R. A. Abrams, S. Kornblum, C. E. Wright, J. E. Smith, Optimality in human motor performance: Ideal control of rapid aimed movements. Psychol. Rev. 95, 340–370 (1988). 3406245 10.1037/0033-295x.95.3.340

[28] D. Elliott ., Goal-directed aiming: Two components but multiple processes. Psychol. Bull. 136, 1023–1044 (2010). 20822209 10.1037/a0020958

[29] C. M. Harris, D. M. Wolpert, Signal-dependent noise determines motor planning. Nature 394, 780–784 (1998). 9723616 10.1038/29528

[30] D. F. Gucciardi, J. A. Dimmock, Choking under pressure in sensorimotor skills: Conscious processing or depleted attentional resources? Psychol. Sport Exerc. 9, 45–59 (2008).

[31] J. Wang, D. Callahan, B. Goldfine, Choking under pressure in competition and psychological intervention approaches. Strength Condit. J. 25, 69–75 (2003).

[32] C. Kidd, B. Y. Hayden, The psychology and neuroscience of curiosity. Neuron 88, 449–460 (2015). 26539887 10.1016/j.neuron.2015.09.010 PMC4635443

[33] J. Gottlieb, M. Cohanpour, Y. Li, N. Singletary, E. Zabeh, Curiosity, information demand and attentional priority. Curr. Opin. Behav. Sci. 35, 83–91 (2020).

[34] R. Ulrich, S. Mattes, Does immediate arousal enhance response force in simple reaction time? Q. J. Exp. Psychol. A 49, 972–990 (1996). 8962543 10.1080/713755672

[35] E. A. Witte, R. T. Marrocco, Alteration of brain noradrenergic activity in rhesus monkeys affects the alerting component of covert orienting. Psychopharmacology (Berl.) 132, 315–323 (1997). 9298508 10.1007/s002130050351

[36] C. Mesagno, D. M. Hill, Definition of choking in sport: Re-conceptualization and debate. Int. J. Sport Psychol. 44, 267–277 (2013).

[37] R. Cools, M. D’Esposito, Inverted-U-shaped dopamine actions on human working memory and cognitive control. Biol. Psychiatry 69, e113–e125 (2011). 21531388 10.1016/j.biopsych.2011.03.028 PMC3111448

[38] G. Aston-Jones, J. D. Cohen, An integrative theory of locus coeruleus-norepinephrine function: Adaptive gain and optimal performance. Annu. Rev. Neurosci. 28, 403–450 (2005). 16022602 10.1146/annurev.neuro.28.061604.135709

[39] G. F. Elsayed, A. H. Lara, M. T. Kaufman, M. M. Churchland, J. P. Cunningham, Reorganization between preparatory and movement population responses in motor cortex. Nat. Commun. 7, 13239 (2016). 27807345 10.1038/ncomms13239 PMC5095296

[40] A. Afshar ., Single-trial neural correlates of arm movement preparation. Neuron 71, 555–564 (2011). 21835350 10.1016/j.neuron.2011.05.047 PMC3155684

[41] L. P. Sugrue, G. S. Corrado, W. T. Newsome, Matching behavior and the representation of value in the parietal cortex. Science 304, 1782–1787 (2004). 15205529 10.1126/science.1094765

[42] M. R. Roesch, C. R. Olson, Impact of expected reward on neuronal activity in prefrontal cortex, frontal and supplementary eye fields and premotor cortex. J. Neurophysiol. 90, 1766–1789 (2003). 12801905 10.1152/jn.00019.2003

[43] M. R. Roesch, C. R. Olson, Neuronal activity related to reward value and motivation in primate frontal cortex. Science 304, 307–310 (2004). 15073380 10.1126/science.1093223

[44] M. L. Platt, P. W. Glimcher, Neural correlates of decision variables in parietal cortex. Nature 400, 233–238 (1999). 10421364 10.1038/22268

[45] K. Louie, L. E. Grattan, P. W. Glimcher, Reward value-based gain control: Divisive normalization in parietal cortex. J. Neurosci. 31, 10627–10639 (2011). 21775606 10.1523/JNEUROSCI.1237-11.2011 PMC3285508

[46] A. Ramakrishnan ., Cortical neurons multiplex reward-related signals along with sensory and motor information. Proc. Natl. Acad. Sci. U.S.A. 114, E4841–E4850 (2017). 28559307 10.1073/pnas.1703668114 PMC5474796

[47] P. Ramkumar, B. Dekleva, S. Cooler, L. Miller, K. Kording, Premotor and motor cortices encode reward. PLoS One 11, e0160851 (2016). 27564707 10.1371/journal.pone.0160851 PMC5001708

[48] B. T. Marsh, V. S. A. Tarigoppula, C. Chen, J. T. Francis, Toward an autonomous brain machine interface: Integrating sensorimotor reward modulation and reinforcement learning. J. Neurosci. 35, 7374–7387 (2015). 25972167 10.1523/JNEUROSCI.1802-14.2015 PMC6705437

[49] W. Schultz, P. Dayan, P. R. Montague, A neural substrate of prediction and reward. Science 275, 1593–1599 (1997). 9054347 10.1126/science.275.5306.1593

[50] A. Lak, W. R. Stauffer, W. Schultz, Dopamine prediction error responses integrate subjective value from different reward dimensions. Proc. Natl. Acad. Sci. U.S.A. 111, 2343–2348 (2014). 24453218 10.1073/pnas.1321596111 PMC3926061

[51] P. S. Chen ., Nonlinear effects of Dopamine D1 receptor activation on visuomotor coordination task performance. Cereb. Cortex 30, 5346–5355 (2020). 32483622 10.1093/cercor/bhaa116

[52] M. Usher, J. D. Cohen, D. Servan-Schreiber, J. Rajkowski, G. Aston-Jones, The role of locus coeruleus in the regulation of cognitive performance. Science 283, 549–554 (1999). 9915705 10.1126/science.283.5401.549

[53] A L. Smoulder ., Data for Monkeys exhibit a paradoxical decrease in performance in high-stakes scenarios. Figshare. 10.6084/m9.figshare.13547435. Deposited 8 April 2021. PMC8536322 34426504

