# Supplementary material for: Monkeys exhibit a paradoxical decrease in performance in high-stakes scenarios
Source: Proc Natl Acad Sci U S A. 2021 Aug 23;118(35):e2109643118. doi: 10.1073/pnas.2109643118 (PMC8536322; doi:10.1073/pnas.2109643118)
Supplement: Supplementary File [file pnas.2109643118.sapp.pdf]

PNAS
www.pnas.org

Supplementary Information for
Monkeys exhibit a paradoxical decrease in performance in high-stakes scenarios.

Adam L. Smoulder†, Nick P. Pavlovsky†, Patrick J. Marino†, Alan D. Degenhart, Nicole T. McClain, Aaron P. Batista*, Steven M. Chase*

† Joint lead authors. See Author Contributions section for more information.
* Joint senior authors. See Author Contributions section for more information.

Corresponding authors: Steven M. Chase, Aaron P. Batista
Email: schase@andrew.cmu.edu, aaron.batista@pitt.edu

This PDF file includes:

SI Materials and Methods
Figures S1 to S8
Tables S1 to S4
SI References

SI Materials and Methods

National Football League Field Goal Success Rate Analysis. We examined success rates of field goals in the NFL under "pressure," here defined as a kick taken with less than 2 minutes remaining in regulation and where the team in possession is between 3 and 0 points behind the opposing team (i.e., where a success would tie the game, at minimum). Data was taken from the 2009-2019 regular season play-by-play data table provided by NFLscrapR (downloaded from https://github.com/maksimhorowitz/nflscrapR). Field goal attempts listed as "blocked" were ignored. Only kicks between 40 and 55 yards were analyzed, as this range was the best match to the overall success rate of our monkeys at the speed + accuracy task. A binomial proportion test was used to determine if success rates were significantly lower when there was pressure. We found a significant ($p < 0.01$) detrimental effect of pressure. To ensure that "pressure" kicks were not longer than the non-pressure comparison, we ascertained the mean and median kick distances for the data. They were nearly identical: all kicks ([mean, median]) were [46.46,46] yards, while pressure kicks were [46.64,46] yards. Other binning methods containing similar numbers of attempts (e.g. quartiles, 40-60 yards, etc.) yielded similar results.

Equipment. During experiments, the monkey sat in a primate chair with his head braced facing a mirror ~8 cm in front of his eyes to view a computer monitor displaying task events. The LCD monitor had a 144 Hz refresh rate. A photodiode was used to measure the timing of the visual display, which was used in analyses to compensate for the lag of the monitor. The monkey performed all tasks by making hand movements (right arm for Monkeys N and E, left arm for Monkey F) in the open space in front of him. The working hand and arm were unrestrained. The hand was not visible to the monkey during experiments, as it moved in the space behind and below the mirror. Hand position was tracked via an LED marker attached to the monkey's index finger (120 Hz sampling rate, nominal resolution <1 mm; PhaseSpace, San Leandro, CA). The monkey's hand movements corresponded to a cursor position displayed to the animal on the LCD monitor. The software environment was calibrated such that 1 cm of lateral hand displacement corresponded to 1 cm of cursor movement.

Speed + Accuracy Task. To succeed at the speed + accuracy task, the monkey had to reach quickly to place the cursor within a small target. The sequence of events in this task is described here and shown Figure 1A. All task parameters for each subject are shown in Table S1 and will be referenced here with quotation marks, with values specified here only for parameters constant across animals. Each trial began with the appearance of a green circle (colored pale blue in figures for visual clarity) in the center of the screen ("Center target diameter"). To initiate a trial, the animal moved a circular red cursor ("Cursor diameter," 6 mm) fully within the central target (i.e. the entire cursor needed to be fully enclosed within the target to continue with the trial). After holding the cursor within the central target for a short period ("Center hold before target onset"), a reach target appeared ("Reach target distance from center," 85 mm). The reach target remained visible while the animal held the cursor in the central target for a variable delay period ("Delay period lengths"). The delay period was drawn from a uniform distribution for each trial from the values shown in Table S1. At the end of the delay period, the central target disappeared, signaling the animal to reach to the cued target. The animal had a limited time to acquire the reach target ("Reach period maximum length"). Once the animal acquired the reach target, he had to hold the cursor fully within the target for 400 ms ("Target hold time requirement"). Upon successful completion of the target hold, the animal received a water reward.

Studies of choking under pressure in humans show that it is most salient for sufficiently difficult tasks (1). However, we reasoned that if we made the task too difficult, the monkeys would give up and stop working. Thus, we adjusted the parameters of the task such that the animals performed at an overall success rate of around 70% before introduction of the Jackpot reward cue (see *Animal Training* below). To do this, we fine-tuned the reach time ("Reach period maximum time") and the size of the reach target ("Reach target diameter"). These parameters required the animals to reach quickly and accurately to the targets.

There were two potential reach targets for Monkeys N and F: left or right. For Monkey E, the target could appear at one of eight locations equally spaced 45° apart, starting at the horizontal ("Number of reach target locations" and "Reach target angle descriptions"). One target appeared on each trial, selected at random. The appearance of the reach target conveyed the potential reward size for successfully completing the trial based on either the inscribed shape's form or color ("Small," "Medium," "Large," and "Jackpot reward cue"). There were 4 potential reward sizes in this task ("Small," "Medium," "Large," and "Jackpot reward size"). We displayed the Jackpot reward on 5% of trials, and the Small, Medium, and Large rewards were evenly divided among the remaining 95% of trials ("Small," "Medium," "Large," and "Jackpot reward frequency"). We designed the reward percentages in this way primarily as a balance between maximizing Jackpot reward size and the number of trials that would be completed within a session before the animals became too satiated.

We initially performed 9 sessions with Monkey E using the same reward values as Monkeys N and F. While choking occurred (Large success rate: 73.7%, Jackpot success rate: 46.5%, significantly different by a binomial proportion test: $p < 10^{-10}$), we did not see any significant improvement from Small to Large rewards (Small: 73.0%, difference from Large $p = 0.61$). This motivated us to change the Small and Large reward values from 0.1 and 0.3 mL to 0 and 0.4 mL. The 11 sessions collected with these reward sizes were analyzed further.

The subject could fail by leaving the center before the go cue, not acquiring the reach target in time, or exiting the reach target before 400 ms had elapsed (see "Task Failure Modes" below). When a trial failed, the cursor froze at the point of failure for 300 ms to provide the animal with visual feedback about his failure mode. To encourage the animals to perform all trials equally well (even those with the smallest rewards), when the animals failed a trial, they had to perform an extra unrewarded reach to a randomly selected target location before a new, rewarding trial would be initiated. These unrewarded trials did not end until the animal completed them.

Two Target Choice Task. We verified the animals' understanding of the reward cues through a two-target choice task that was performed each day after experiments with the speed + accuracy task were complete. The reach time, reach target size, and other parameters were the same in this task as in the speed + accuracy task. However, instead of one target appearing per trial, two targets were shown, always 180° apart. Figure S1A shows possible target configurations, with connecting lines showing pairs of targets simultaneously shown. Each of the two reach targets cued a reward size of the same size as in the speed + accuracy task. The animals could reach to either target to receive the corresponding reward. To evaluate if the animals had biases towards any particular target direction, some trials presented the same reward cues at both locations. For illustration, we colored the main directions of bias for each subject in blue, with the opposing targets being colored red (Fig. S1A). Monkey N chose the higher reward on 88.3% of choice trials, and exhibited a strong leftward bias (Fig. S1B, left). We found that nearly all of his lower-value choices were towards the left (72/73, 98.6% of wrong choices) and often involved the next lower-valued reward (67/73, 91.9% of wrong choices). Monkey F chose the higher reward on 99.8% of choice trials (Fig. S1B, center), and Monkey E chose the higher reward on 98.6% of choice trials (Fig. S1B, right).

Jackpot Reward Magnitude and Rarity Dissociation Experiments. In the speed + accuracy task, the Jackpot reward was larger in magnitude than the other rewards, and it appeared less frequently. Thus, it could be that choking was caused by the Jackpot reward's magnitude, its rarity, or both. To examine if reward magnitude or rarity alone was sufficient to induce choking, we ran two control experiments with Monkey E. Each of these control experiments were nearly identical to Monkey E's version of the speed + accuracy task, with key differences outlined below.

In the first control experiment, we sought to determine whether reward rarity was sufficient to induce choking. In order to do so, we introduced a fifth reward into the speed + accuracy task, the "Rare-Large" reward (Fig. 2A, Table S1 column "Monkey E, rare-large sessions"), which had the same magnitude as the Large reward ("Additional reward size") but was as rare as the Jackpot reward ("Additional reward frequency"). We assigned a unique cue to the Rare-Large reward ("Additional reward cue") and verified Monkey E's understanding of this new cue by including it in the two-target

choice task (Fig. S1C). We used the following reward distribution for this control experiment: Small on 31.6% of trials, Medium on 31.6% of trials, Large on 26.6% of trials, Rare-Large on 5% of trials, and Jackpot on 5% of trials (Fig. 2A, top). This choice of distribution ensured that the overall expected reward for the control experiment matched that of the speed + accuracy task. We performed 9 sessions of this experiment.

In the second control experiment, we asked whether reward magnitude was sufficient to induce choking. To do so, we again modified the original speed + accuracy task by adding a different fifth reward. This new "Common-Jackpot" reward (Fig. 2B, Table S1 column "Monkey E, common-jackpot sessions") had the same magnitude as the Jackpot reward ("Additional reward size") but appeared as frequently as the non-Jackpot rewards ("Additional reward frequency"). We assigned a unique cue to the Common-Jackpot reward ("Additional reward cue") and verified Monkey E's understanding of the new cue by including it in the two-target choice task (Fig. S1D). Because both the Jackpot and Common-Jackpot cues were very large in magnitude, it was difficult to match the overall expected reward in this control experiment to that of the speed + accuracy task. Instead, we chose a distribution in which all rewards other than the Jackpot occurred with equal probability: Small, Medium, Large, and Common-Jackpot rewards on 23.75% of trials each, and Jackpot on 5% of trials. We performed 6 sessions of this experiment.

Precision Task. In addition to the speed + accuracy task, Monkey F performed a precision task (Fig. S2A, in Table S1 column "Monkey F, precision task"). The animal was presented with a curved path to follow to reach the target, located 100 mm away from the center ("Reach target distance from center"). The path started at the center target ("Center target diameter," 9 mm) and ended at the reach target ("Reach target diameter," 24 mm). The paths had a single inflection in either the clockwise or counterclockwise direction and were 12 mm in width ("Reach target location description"). The center of the cursor had to stay within the path in order to successfully complete the trial. This task emphasized precise movements rather than speed. The allotted reach time was 2000 ms, the delay period lasted between 550 and 1050 ms (drawn from a uniform distribution at 100 ms intervals), and the reach target was held for 400 ms. The reward sizes and cue colors were the same as in the speed + accuracy task for Monkey F.

For the precision task, in addition to different reward sizes, we also varied the "punishments" that happened after a failed trial. We had four levels of punishment that were also cued at the reach target by the shape inscribed within the target (where color cued the reward size). The punishments corresponded to the number of unrewarded reaches the animal had to make after a failed trial prior to initiating a new rewarding trial (between 1 and 8). We did not see any change in behavior associated with the different punishments; therefore, we have combined the data analyzed here across all different punishment conditions. Before settling on the task parameters stated above, we initially ran 28 sessions using a smaller jackpot reward size (Mini-Jackpot; for Table S1, uses "Jackpot" rows) of 0.8 mL instead of 2.0 mL (Fig. S3C, in Table S1 column "Monkey F, precision task, mini-jackpot sessions"). Notably, neither the threat of a maximum punishment (8 unrewarded reaches) nor a smaller Jackpot reward magnitude led to choking.

Animal Training. Prior to beginning training on the speed + accuracy task, animals were already trained at a delayed center-out reaching task in which reward magnitude was constant. Our training procedure for the speed + accuracy task had two phases. In the first phase, we familiarized animals with the Small, Medium, and Large reward cues and titrated task difficulty. In the second phase, we familiarized them with the Jackpot cue. Each session during training consisted of a block of trials of the speed + accuracy task followed by a block of trials of the two-target choice task. All data reported in this manuscript are from a period after the animals had demonstrated proficient understanding (defined below) of each reward cue's value relative to the others.

During the first phase of training, only the Small, Medium, and Large rewards were used in both tasks. We increased the difficulty of the speed + accuracy task by adjusting the reach time and target size until animals were approximately 70% successful. We assessed the animals' understanding of the reward cues using the two-target choice task. Once the speed + accuracy

task was sufficiently difficult and animals showed proficient understanding of the reward cues (i.e., the higher reward was selected at least 90% of the time), we began the second phase of training. In the second phase, Jackpot rewards were introduced in both tasks. When animals demonstrated proficient understanding of the Jackpot cues in the two-target task, we began accepting data for analysis the next session.

Task failure modes. While choking can be observed as an inverted-U shape in overall success rates as a function of reward, this alone does not identify whether or not the failures at the highest and lowest levels of reward are occurring in a similar fashion. Each unsuccessful trial could be categorized into one type of failure, as described below. Performing this classification enabled us to evaluate the trends of frequency for each specific failure mode as reward size increased (Fig. 4, S5). We identified five primary different ways that the animals failed based on the speed + accuracy task design.

Before the go cue occurred, there were two main ways failure could occur: “false starts” and “delay drifts” (Fig. S5A, left). A false start occurred when it appeared that a full reach was beginning to be executed before 100 ms after the go cue occurred (assuming a minimum reaction time of 100 ms). A false start was defined if two criteria were met: (1) the cursor moved more than 1.75 times the start target radius within 150 ms of exiting, and (2) the cursor was within a 45° arc of the target direction. Delay drifts were failures where the cursor appeared to slowly drift out of the target and were evaluated as failures before 100 ms after the go cue where the cursor was still within 1.75 times the start target radius of center at 150 ms after exiting. We validated this categorization of failure modes by making a scatterplot of distance from center target at 150 ms after failure versus speed at time of failure. In these plots, we saw a clear separation of two clusters, with low values indicating delay drifts, and high values indicating false starts. Minor changes to parameters (i.e., using 1.5 or 2 times start target radius) did not significantly affect this classification.

After the go cue, the animals had a limited time to reach the end target. There were two main ways failures could happen during the reach: “overshoots” and “undershoots” (Fig. 4A). An overshoot was classified in one of two cases: (1) the subject reached beyond the center of the reach target without entering it (i.e, their hand moved a sufficient distance, but they missed the target), then did not have sufficient time to make a corrective reach back towards it (187 total, 13.6% of overshoots), or (2) the subject reached through the reach target without stopping (1193 total, 86.4% of overshoots), defined as spending less than 250 ms within the target. Note that it is possible to overshoot the target without going past the center distance if the cursor scrapes through the near edge. We also considered other classifications using different thresholds of speed to determine if the reach was still continuing in the reach target (i.e., cursor speed > 0.05 m/s, 0.02 m/s, 10% of peak speed) and achieved similar results. An undershoot was classified as any attempted reach that did not make it to the target in time. This includes both trials where time expired mid-reach (1619 total, 75.2% of undershoots) and reaches that landed short of the target with insufficient time for corrective movements (533 total, 24.8% of undershoots). We selected 500 total example endpoints from one subject to illustrate these different statuses (Fig. 4A).

Once the subject had landed within the reach target, they had to hold for a short period of 400 ms before a reward was earned. The main failure method in this epoch was “target hold drifts” (Fig. S5C). A failure was classified as a target hold drift if the cursor left the reach target with an exit speed of less than 0.1 m/s.

These five failure modes along with successes account for 98.8% of all trials (18764/18989). The remaining 217 trials were excluded from analyses and are composed of “quit-outs” (71 trials), “no-attempts,” (124 trials), “wild” reaches (22 trials), and “early-returns” (8 trials). Each excluded trajectory was manually examined to validate classification. Quit-outs were classified as trials where the subject reached in a direction more than 45° away from the reach target, either before or after the go cue. Trials of these failure modes were excluded from analysis due to their infrequency and/or the evident lack of an intent at performing the task. As each of these failure modes occurred with greatest frequency for Small and/or Jackpot rewards, we note that excluding these trials yields

a conservative interpretation of our results where we indicate Small and Jackpot-reward trials having lower success rates than Large reward trials (Fig. 1B).

Success and Failure Rate Analysis. We analyzed success rates as a function of reward size. For main results, we did this for all sessions combined within an experiment (Fig. 1, 2, S2B, S2C). For supporting results, we also analyzed success rates for each individual session (Table S2, Fig. S3A), for the first or second half of sessions (Fig. S3B), and also for a subset of late sessions of the precision task (Fig. S2B). We calculated mean success rates and standard error bars using a bootstrapping method as described in Materials and Methods. For main results, we pooled across sessions as opposed to taking the mean of the results from individual sessions.

We also analyzed the rates of different failure modes as a function of reward (Fig. 4, S5). When analyzing failure modes, we only considered trials in which the monkey had made it to the epoch of the task where the failure mode occurred, and we considered an "epoch success" as any trial that did not fail within that period. More specifically: For the delay period failure mode analyses (Fig. S5A), all trials were used, and an epoch success was any trial that did not leave the center target before 100ms after the go cue appeared (accounting for a minimum reaction time of 100 ms). For the reach period failure mode analyses (Fig. 4), only trials that successfully completed the delay epoch were used, and an epoch success was any trial where the reach successfully landed in the end target (i.e., no undershoot or overshoot; see *Task Failure Modes*). For target hold period failure mode analyses (Fig. S5C), we only used trials that were epoch successes for the reach period, and a trial was classified as an epoch success if the cursor was within the reach target for 400 ms; this is the same as successfully completing the trial. We calculated error bars and significant differences between failure rates for different rewards in the same manner as for success rates described in Materials and Methods.

We assessed post-hoc if the relative target difficulty contributed to success rate (Fig. S7). We split the data within each subject by reward cue, target location, and session. We then divided the session-target combinations (e.g., Session 2, leftward target) into quartiles based on Medium reward success rate. We then calculated success rate as a function of reward within the "easiest" (highest Medium success rate) and "hardest" (lowest Medium success rate) quartiles for comparison. We also sought to determine if the previous trial's success or failure affected choking (Fig. S8). To do this, we simply conditioned the data on reward cue and the status (success or failure) of the previous trial, then calculated success rates.

Kinematic Analysis. We analyzed the animals' reach kinematics in the speed + accuracy task to determine how reaching behavior changed as a function of reward. We calculated four metrics: reaction time (Fig. S5B), homing time (Fig. 3A, C), peak speed (Fig. S5B), and ballistic endpoint prediction (Fig. 3A, B). For all of these calculations, only trials that completed the delay epoch were considered (i.e., false starts and delay drifts are removed). Reaction time was calculated as the time between the go cue and when the cursor exited the location of the central target. We saw no qualitative difference in trends if we used a metric based on cursor velocity instead (i.e., defining 20% of peak speed as the end of reaction time). We found the distributions for reaction time within each reward size to have heavy tails, allowing outliers to heavily bias the mean. To avoid having our interpretation of results influenced by this, we instead use the median. We calculated the median and its standard error using a bootstrapped distribution of the median's mean and standard deviation, as described in Materials and Methods, bias corrected as specified in (2). We also plotted the distributions of reaction time conditioned on reach success or failure mode in violin plots (Fig. S5B).

The goal of using homing time was to gauge if the animals were spending more time near or approaching the reach target as reward size increased (Fig. 3A). To evaluate this, we had to select three parameters: (1) where homing time begins, (2) where homing time ends, and (3) for reaches that fail due to running out of time, how much longer after the failure time is permitted to achieve the endpoint of homing time. For the first, we selected ⅔ of the distance to the reach target, though we found similar trends across rewards if we used other starting locations (i.e., halfway to target,

¾ distance to reach target). We avoid using location of peak speed as a starting point, as it changes on a trial-by-trial basis and we are seeking to evaluate the time taken to cover a specific distance. For the latter two parameters, in an ideal scenario, we would select target entry as the endpoint of homing time and 0 ms after failure time. However, this would require the removal of all undershoot (and some overshoot) failure trials, where many of them are nearly about to acquire the reach target when time expires. We also found that this problem disproportionately affected Jackpot reward trials due to the higher incidence of undershoot failures (Fig. 4B). To strike the balance of including as many of these as possible while still maintaining the assumption that failure information does not affect the reach trajectory within 150 ms of its occurrence, we chose 1 mm away from the reach target as the endpoint for homing time and allowed 150 ms after time had expired to achieve this distance. This excluded 1.2% of all trials that made it to the reach epoch (207/17604 trials), and within that, 3.4% of Jackpot trials (28/823 trials). We tried a variety of other parameter values and found the same trends present that support the conclusions in the text, including all combinations of [0, 0.5, 1, 1.5, 2] mm away as the endpoint for homing time, and [0, 50, 100, 150, 200] ms after failure being permitted for time of acquiring the endpoint. Like with reaction time, the homing time distribution was heavy-tailed, so we calculated the median and standard error of the median over trials within each reward size (Fig. 3C). To ensure the observed homing time trends were not simply a result of slower reaching overall, we also looked at homing time as a fraction of the full reach time, defined as the time elapsed from go cue until the end of homing time (Fig. S4).

To obtain peak speed (Fig. S5B) and ballistic endpoint predictions (Fig. 3A, B), we first calculated the horizontal and vertical cursor velocity at each time point after the go cue until failure or reach target acquisition. We then smoothed these traces with a 4th order zero-phase FIR low-pass filter (cutoff frequency = 15 Hz). The peak speed for each trial was the maximum value across time of the square root of the sum of the squared velocity traces (i.e., the maximum of the velocity magnitude trace). To calculate the ballistic endpoint prediction for a given trial, we took the section of the velocity traces from go cue until peak speed and mirrored them. This produced symmetric, bell-shaped velocity/speed profiles like those typically associated with rapid point-to-point reaches (3, 4) though not typically seen in the speed + accuracy task, presumably due to the corrective movements that are associated with small reach target sizes (5, 6). We integrated these to predict the cursor location at the end of these profiles and called this endpoint the ballistic (reach) endpoint prediction. We call this a "ballistic" prediction under the assumption that feedback-driven corrective movements are not yet incorporated during this first phase of the reach (7–10). Because distributions for both peak speeds and ballistic endpoint predictions appeared normally distributed conditioned on reward, we show mean and standard error of the mean. Like with reaction time, we made violin plots for peak speed conditioned on reach success or failure mode (Fig. S5B).

Data and materials availability. All code used for preprocessing and analysis is available at https://github.com/adam-smoulder/MonkeysChokeUnderPressure_behavior. Data is available at DOI: 10.6084/m9.figshare.13547435.

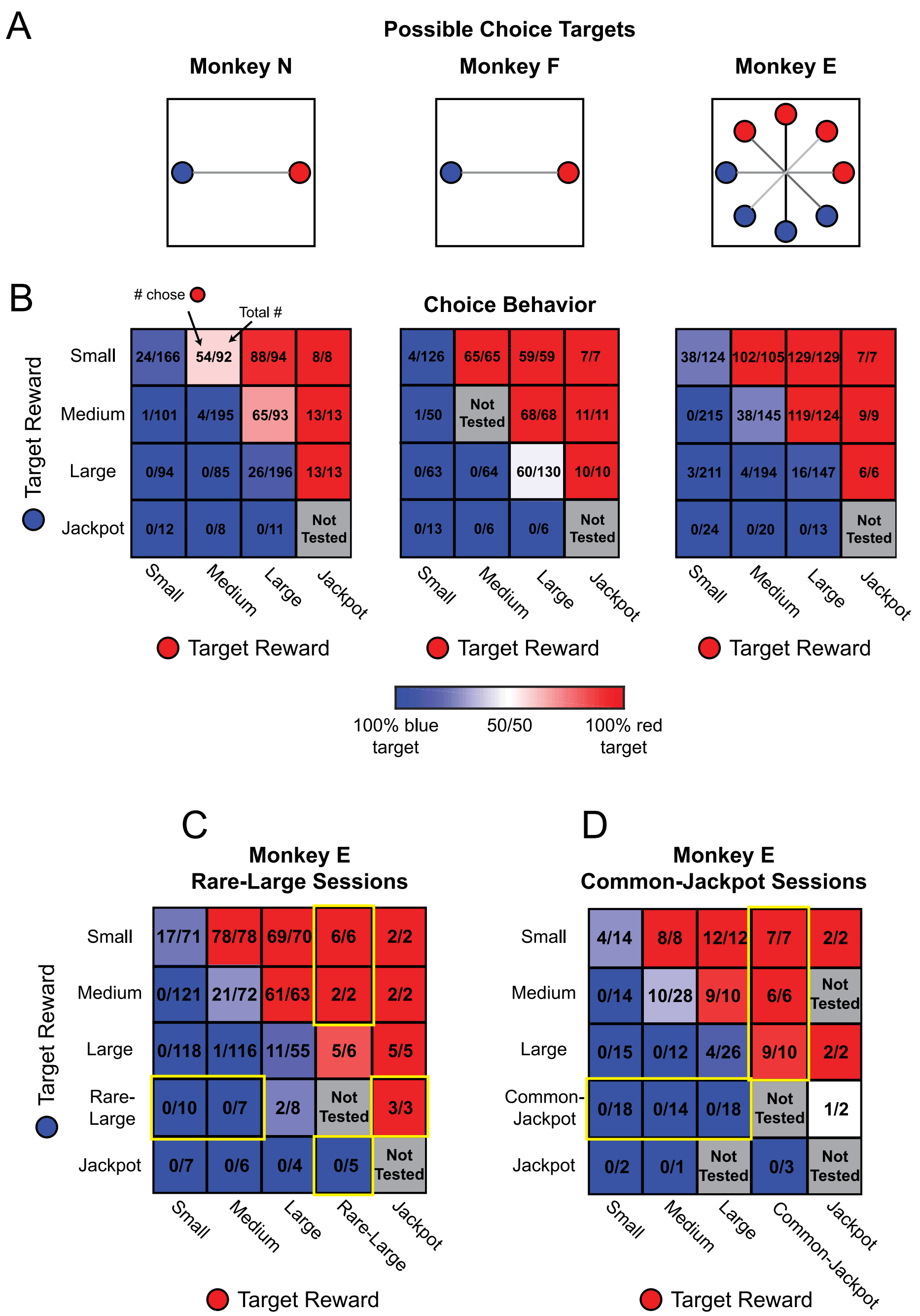

Fig. S1. Two-target choice task behavior. (A) To determine if the monkeys understood the value of each reward cue relative to the others, we designed a choice task where the animals reached towards one of two targets shown simultaneously: one at a red location, and one at a blue location. Note that these were not the actual colors of the targets and are just used for representation of locations in this figure. For a given trial, the presented targets were always 180 degrees apart. Target location pairs are connected with grayscale lines. Reward cues were the same as those used in the speed + accuracy task (see Table S1 for details). (B) Choice behavior in each animal. Monkey N (left) chose the higher reward on 88.3% of trials. The trials with matching rewards (main diagonal of grid) revealed a leftward (blue) bias to his choices, which may account for his incorrect choices when the higher reward was at the rightward target (98.6% of wrong choices are in direction

of bias). Monkey F (center) chose the higher reward on 99.8% of trials. This animal also had a bias to the left target, but it was not as strong as Monkey N and did not affect his choice behavior. Monkey E (right) chose the higher reward on 98.6% of trials and exhibited a down-leftward (blue) bias. We note that for all animals, the Jackpot reward was selected at every opportunity possible. (C) Monkey E's choice behavior for sessions including the Rare-Large cue. Yellow boxes indicate key comparisons for determining if the relative value of the Rare-Large cue has been learned. Of these, Monkey E made correct selections 100% of the time. (D) Same as (C) but for the Common-Jackpot cue. Monkey E selected correctly for 98.6% of trials that Common-Jackpot was greater than the other non-Jackpot rewards, boxed in yellow.

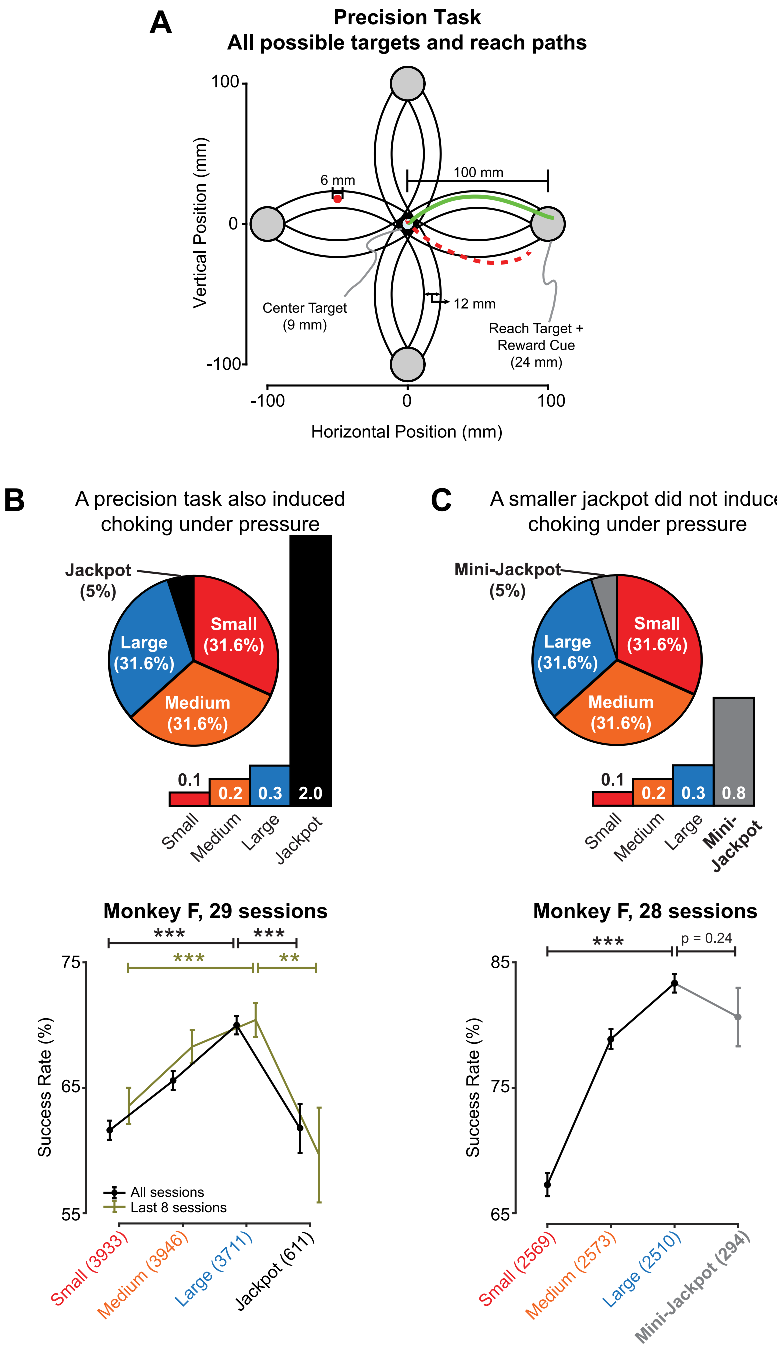

Fig. S2. A precision task also induces choking under pressure. (A) Precision task layout. One monkey (F) performed a task that followed the same timeline as shown in Figure 1A, though instead of there being a stringent reach time requirement, the cursor had to remain within a designated path. All possible targets and reach paths are shown, with each trial indicating a single path, reach target, and reward cue at the beginning of the delay period. Target diameters are indicated in parentheses. Moving the cursor outside of the reach path would cause the trial to fail. An example successful (green) and failed (red) reach trajectory are shown. (B) The reward cue distributions and magnitudes matched that of the speed + accuracy task for Monkey F (top). Average success rate (error bars, S.E.) versus cued reward size combined across all 29 sessions is shown (bottom,

black). Stars indicate significant differences between mean success rates evaluated using a binomial proportion test: ** = $p < 0.01$, *** = $p < 0.001$. Significance is only shown for Small to Large and Large to Jackpot comparisons for visual clarity (see Table S2 for all comparisons). We also show the success rates for the last 8 sessions to demonstrate that choking did not go away after prolonged exposure to the Jackpot reward (olive). We used 8 sessions to match the number of sessions Monkey F performed for the speed + accuracy. (C) In pilot experiments, we had Monkey F perform the precision task with a smaller, “Mini-Jackpot” reward of 0.8 mL, with the same frequency of occurrence as the Jackpot reward in all other experiments (gray, top). Mean success rate as a function of reward is shown (bottom).

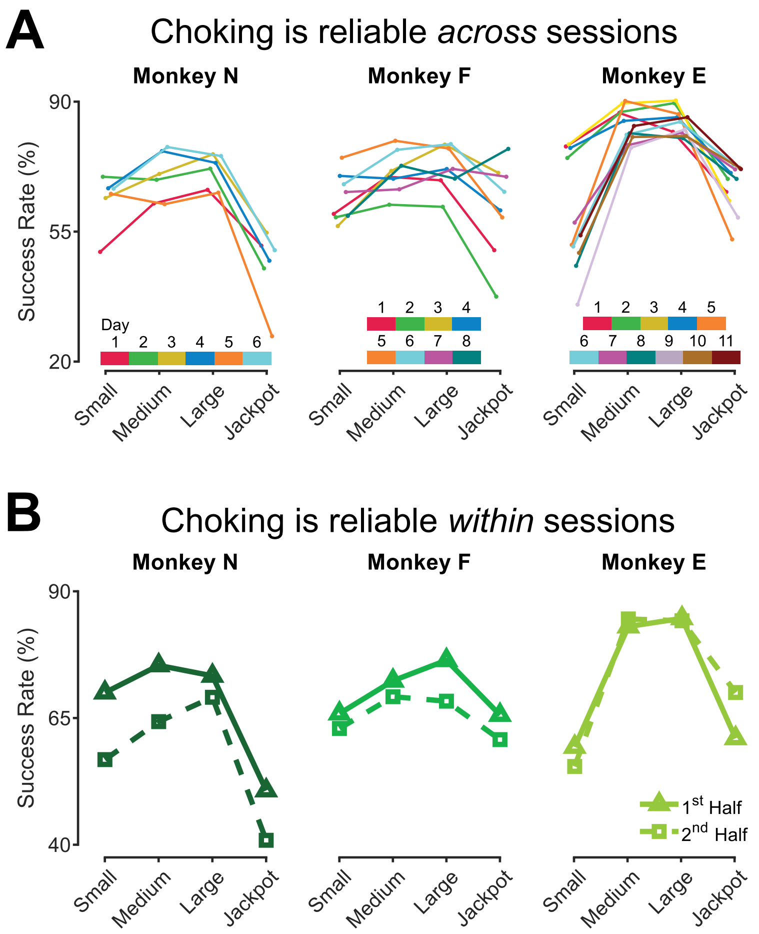

Fig. S3. Choking is reliable across and within sessions. (A) Individual session success rate versus reward size. All three animals showed a decrease in success rate between the Large and Jackpot rewards for multiple sessions. Monkeys N and E exhibited choking under pressure in all sessions. Monkey F choked under pressure in 7 of 8 sessions. Data are jittered on the horizontal axis for visual clarity. (B) Success rate versus reward using only the first (solid, triangles) or second (dashed, squares) half of trials from each session.

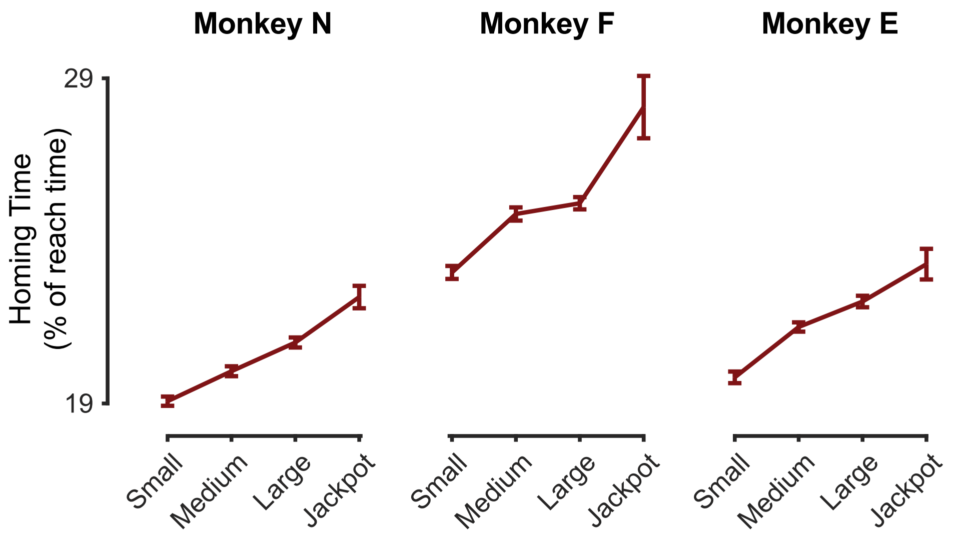

Fig. S4. Percentage of reach epoch spent on homing time (⅔ of reach target distance to 1 mm away) as a function of reward. Medians with standard error bars are shown. "Reach time" is defined as the time from go cue until the cursor achieved 1 mm away from the reach target.

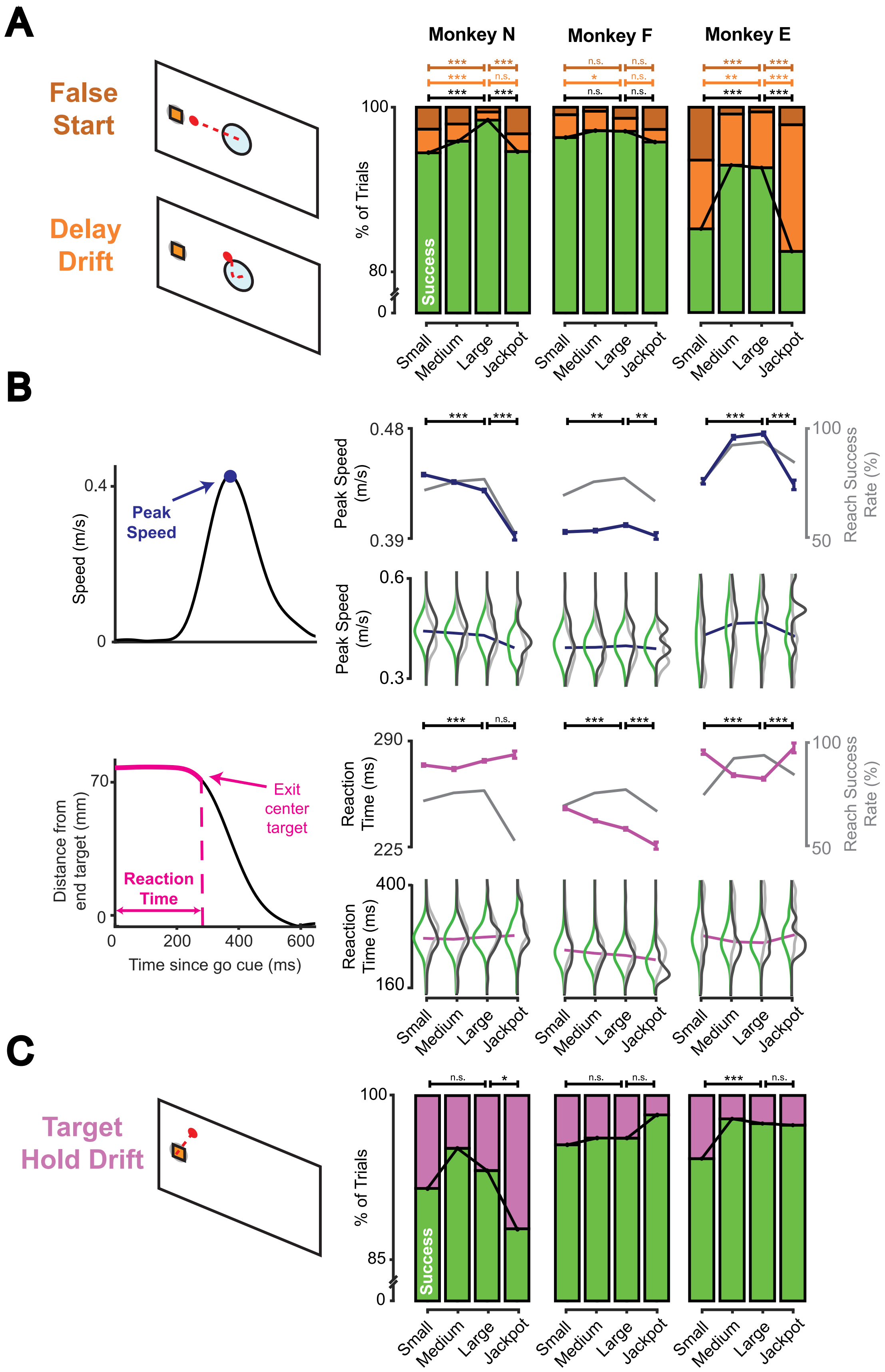

A
False Start
Delay Drift
Monkey N
Monkey F
Monkey E
% of Trials
100
80
0
Success
Small
Medium
Large
Jackpot
B
Peak Speed
Speed (m/s)
0.4
0
Peak Speed (m/s)
0.48
0.39
0.6
0.3
Reach Success Rate (%)
100
50
Distance from end target (mm)
70
Exit center target
Reaction Time
Time since go cue (ms)
200
400
600
Reaction Time (ms)
290
225
400
160
C
Target Hold Drift
85

Fig. S5. Idiosyncratic behaviors contribute to choking. Stars indicate significance: * = $p < 0.05$, ** = $p < 0.01$, *** = $p < 0.001$. (A) In the delay epoch (after target onset, before go cue), subjects can fail by either commencing a full reach early ("False Start," dark orange, top-left) or by slowly drifting out of the target ("Delay Drift," bright orange, bottom-left). Frequencies for each of these failures along with trials that successfully made it into the reach epoch (green) are shown (right). We emphasize that green here indicates trials that successfully complete the delay epoch (no failure before go cue), not necessarily complete trial successes. Significant differences in the rates of each failure mode (or successful delay period) across reward sizes were evaluated using a binomial proportion test. Significance is only shown for Small to Large and Large to Jackpot comparisons for visual clarity. (B) We calculated the peak speed and reaction time for each trial. In the top-right sub-panel, the mean peak speed is shown for each reward size (navy traces). Below are violin plots showing the distribution of peak speeds for successful (green), overshoot (black), or undershoot (gray) trials, with the means for all trials overlaid. In the bottom-right panel are median reaction times for each reward size (pink traces). We used the median, rather than the mean, since reaction time distributions had heavy tails. Below are violin plots of showing the distribution of reaction times for successful (green), overshoot (black), or undershoot (gray) trials, with the medians for all trials overlaid. Error bars for peak speed and reaction time are S.E. of mean and median, respectively. (C) Once subjects successfully landed the cursor within the end target, it was possible to still fail the trial if they allowed the cursor to exit the target before the 400 ms hold has expired ("Target Hold Drift," left, pink). This failure rate is shown as a function of reward, along with the fraction of trials that land within the target and successfully complete the target hold, and thus successfully complete the trial (green). Because successes and target hold drifts cover all trials that acquire the end target, significance calculations for the two are equivalent.

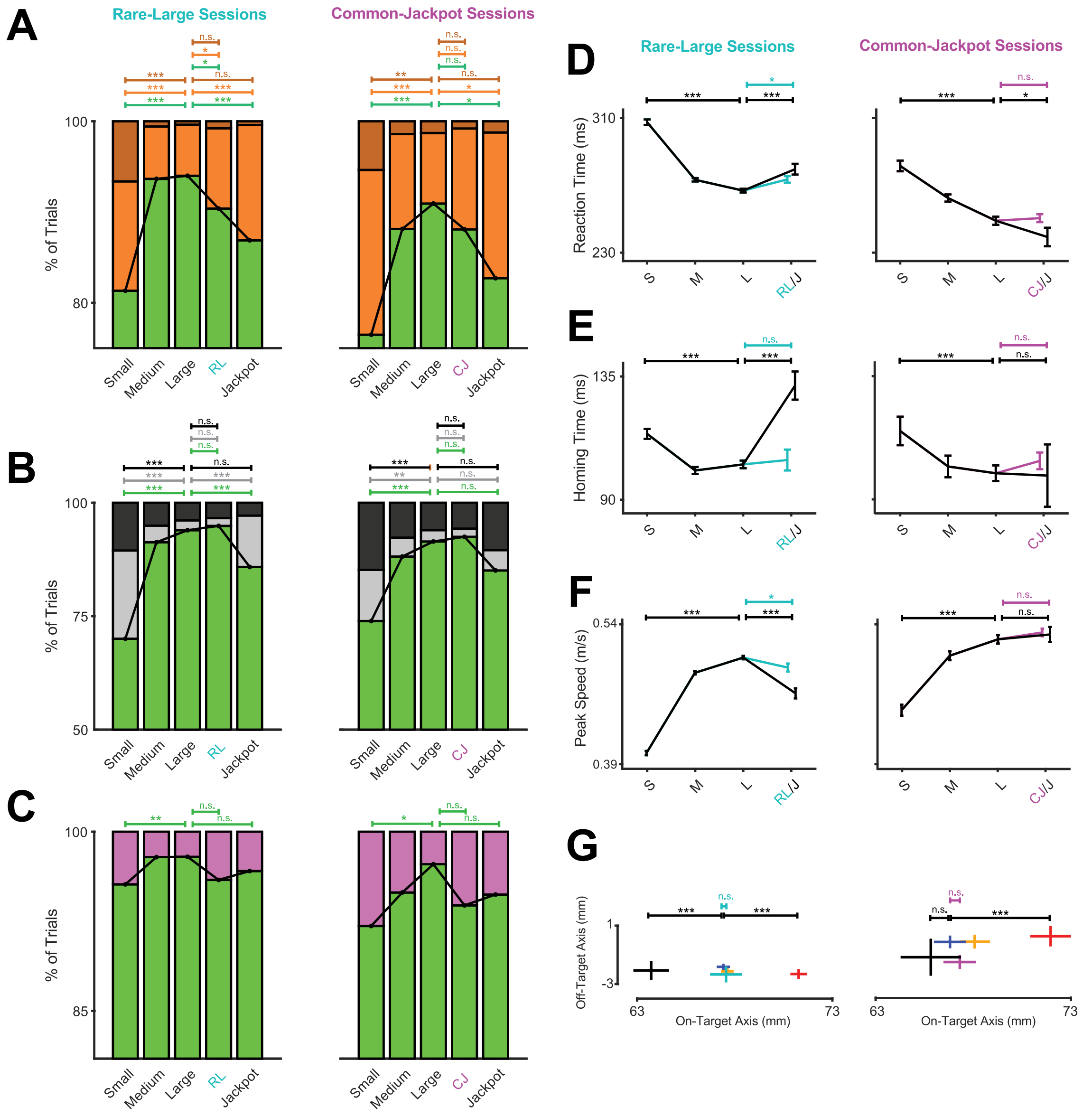

Fig. S6. Additional behavioral results from Monkey E's Rare-Large and Common-Jackpot sessions. In all panels, the Rare-Large session results are in the left subpanel, and Common-Jackpot session results are in the right subpanel. Stars indicate significance as described in Figure 1, with only comparisons shown for Small to Large, Large to Jackpot, and Large to Rare-Large/Common-Jackpot for clarity. A binomial proportion test was used for panels A-C, Mann-Whitney U-test for D-E, and Welch's t-test for F-G. (A) Delay epoch failure and success rates as a function of reward size. Colors follow Figure S5A (dark orange = false start, light orange = delay drift, green = success). Comparisons indicate differences in success rate and the rates of the individual failure modes across reward sizes. (B) Reach epoch failure and success rates. Colors follow Figure 4B (black = overshoot, gray = undershoot, green = success). (C) Target hold epoch failure and success rates. Colors follow Figure S5C (Magenta = target hold drift, green = success). (D) Reaction time versus reward. For panels D-F, Rare-Large (cyan) and Common-Jackpot (Purple) results are shown in the same style as Figure 2, where the black trace shows the trend across the normal rewards. Median +/- SE shown. (E) Homing time versus reward. Median +/- SE shown. (F) Peak speed versus reward. Mean +/- SE shown. (G) Ballistic reach endpoint predictions as a function of reward. Mean +/- SE shown.

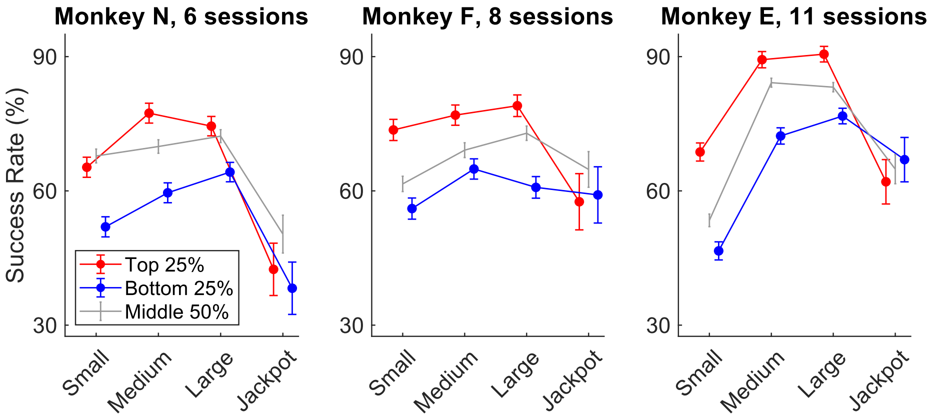

Fig. S7. Animals choke for both easier and harder targets. Trial success rates (SE bars) versus reward are shown for "easier" (red), "harder" (blue), and "intermediate" (gray) session-target combinations. For each animal, we calculated the success rate as a function of reward, target, and session. We then divided the session-target combinations into quartiles based on Medium reward success rate. We compared the "easiest" quartile (red), using the session-targets with the highest Medium success rate, to the "hardest" quartile (blue), using the session-targets with the lowest Medium success rate. The middle 50% success rates are shown as well (gray). Average success rate +/- SE is shown, pooled across the session-targets in the given subset.

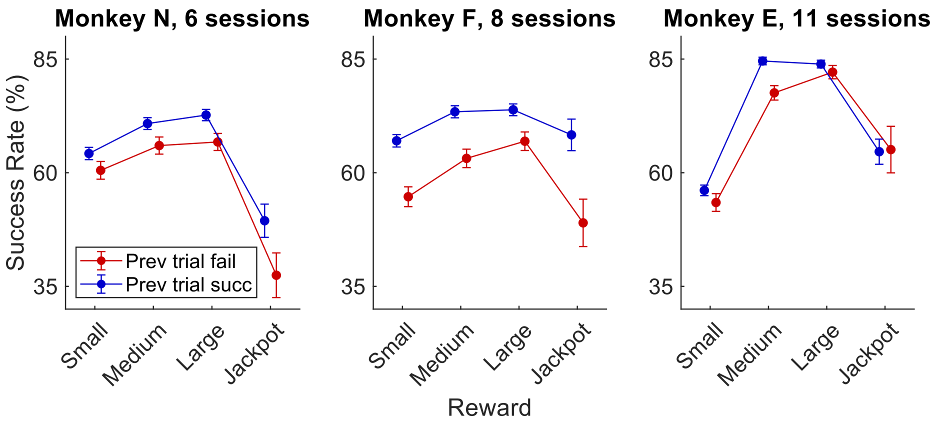

Fig. S8. A previous trial's success or failure had little bearing on choking. Trial success rates are shown for trials following a success (blue) and trials following a failure (red) as a function of reward. Average success rates (and SE) were then calculated using the same bootstrapping procedure as in Figure 1 (see Methods).

Table S1. Task conditions for each set of experiments. Columns labeled solely as “Monkey ___” correspond with the speed + accuracy task. Asterisks (*) are used for Monkey F’s precision task cues to indicate that these were not the only cues used: for the precision task, colors indicated reward size, while the inscribed shape indicated the potential number of “punishment” reaches for failure (see SI Methods).

**Subject Name**	Monkey N	Monkey F	Monkey E	Monkey E, standard reward range	Monkey F, precision task	Monkey F, precision task, mini-jackpot sessions	Monkey E, rare-large sessions	Monkey E, common-jackpot sessions
Number of sessions	6	8	11	9	29	28	9	6
Dates of sessions	2018: 11/26-11/30, 12/3	2018: 11/30-12/1, 12/4-12/9	2019: 11/1, 11/4-11/8, 2020: 6/17-6/18, 6/22-6/24	2019: 10/14-10/18, 10-24-10/25, 10/28-10/29	2018: 2/26-3/2, 3/4-3/5, 3/8-3/17, 3/19-3/24, 3/26-3/30, 4/3	2018: 1/3-1/5, 1/8-1/12, 1/14-19, 1/21-1/26, 1/29-2/2, 2/4-2/5, 2/8-2/9	2020: 6/28-7/1, 7/3, 7/5-7/8	2020: 7/12-7/17
Associated Figures	1,3,4,S1, S3,S4,S5, S7, S8	1,3,4,S1, S3,S4,S5, S7, S8	1,3,4,S1, S3,S4,S5, S7,S8	N/A	S2	S2	2, S1, S6	2, S1, S6
Number of reach target locations	2	2	8	8	4 (each with 2 paths)	4 (each with 2 paths)	8	8
Reach target location descriptions	Right, left	Right, left	45 degrees separated, spaced evenly starting at horizontal	45 degrees separated, spaced evenly starting at horizontal	Targets at right, up, left, down, each with 2 (CW, CCW) 12 mm diameter paths	Targets at right, up, left, down, each with 2 (CW, CCW) 16 mm diameter paths	45 degrees separated, spaced evenly starting at horizontal	45 degrees separated, spaced evenly starting at horizontal
Reach target distance from center	85 mm	85 mm	85 mm	85 mm	100 mm	100 mm	85 mm	85 mm
Reach target diameter	11.4 mm	10.5 mm	14.6 mm	14.6 mm	24 mm	24 mm	14.6 mm	14.6 mm
Center target diameter	17.4 mm	16.7 mm	16.6 mm	16.6 mm	9 mm	9 mm	16.6 mm	16.6 mm
Cursor diameter	6 mm	6 mm	6 mm	6 mm	6 mm	6 mm	6 mm	6 mm
Center hold before target onset	250 ms	250 ms	200 ms	200 ms	250 ms	250 ms	200 ms	200 ms
Delay period lengths (ms)	[250, 350, 450, 550]	[250, 350, 450, 550]	[450, 550, 650, 750, 850, 950]	[450, 550, 650, 750, 850, 950]	[550, 650, 750, 850, 950, 1050]	[550, 650, 750, 850, 950, 1050]	[450, 550, 650, 750, 850, 950]	[450, 550, 650, 750, 850, 950]
Reach period maximum time	625 ms	625 ms	750 ms	750 ms	2000 ms	2000 ms	750 ms	750 ms
Target hold time requirement	400 ms	400 ms	400 ms	400 ms	400 ms	400 ms	400 ms	400 ms
Small reward size	0.1 mL	0.1 mL	0.0 mL	0.1 mL	0.1 mL	0.1 mL	0.0 mL	0.0 mL
Small reward frequency	31.67%	31.67%	31.67%	31.67%	31.67%	31.67%	31.67%	23.75%
Small reward cue					*	*		
Medium reward size	0.2 mL	0.2 mL	0.2 mL	0.2 mL	0.2 mL	0.2 mL	0.2 mL	0.2 mL
Medium reward frequency	31.67%	31.67%	31.67%	31.67%	31.67%	31.67%	31.67%	23.75%
Medium reward cue					*	*		
Large reward size	0.3 mL	0.3 mL	0.4 mL	0.3 mL	0.3 mL	0.3 mL	0.4 mL	0.4 mL
Large reward frequency	31.67%	31.67%	31.67%	31.67%	31.67%	31.67%	26.67%	23.75%
Large reward cue					*	*		
Jackpot reward size	2.0 mL	2.0 mL	2.0 mL	2.0 mL	2.0 mL	0.8 mL	2.0 mL	2.0 mL
Jackpot reward frequency	5%	5%	5%	5%	5%	5%	5%	5%
Jackpot reward cue					*	*		
Additional reward name	-	-	-	-	-	-	Rare-Large	Common-Jackpot
Additional reward size	-	-	-	-	-	-	0.4 mL	2.0 mL
Additional reward frequency	-	-	-	-	-	-	5.00%	23.75%
Additional reward cue	-	-	-	-	-	-		

Table S2. Success rates for individual sessions of the speed + accuracy task and overall performance for all other experiments. Trial numbers are shown below success rates (successes / total). The "stats" columns indicate statistically significant differences between success rates of the current column's reward size versus the other rewards (indicated by color) in the same session/experiment (same row) using a binomial proportion test. Stars indicate significance: * = p < 0.05, ** = p < 0.01, *** = p < 0.001. Experiment names are shown in the first column and match those shown in the columns of Table S1.

		**Small Reward**		**Medium Reward**		**Large Reward**		**Jackpot Reward**	
		Successful/ Total Trials	Stats	Successful/ Total Trials	Stats	Successful/ Total Trials	Stats	Successful/ Total Trials	Stats
Monkey N	Day 1	49.5%<br>163/329	-<br>**<br>***<br>ns	62.6%<br>184/294	**<br>-<br>ns<br>ns	66.2%<br>210/316	***<br>ns<br>-<br>ns	51.2%<br>21/41	ns<br>ns<br>ns<br>-
	Day 2	69.8%<br>210/301	-<br>ns<br>ns<br>***	69.0%<br>211/306	ns<br>-<br>ns<br>***	72.0%<br>236/328	ns<br>ns<br>-<br>***	45.1%<br>23/51	***<br>***<br>***<br>-
	Day 3	64%<br>203/317	-<br>ns<br>**<br>ns	70.5%<br>225/319	ns<br>-<br>ns<br>*	75.8%<br>229/302	**<br>ns<br>-<br>**	54.7%<br>29/53	ns<br>*<br>**<br>-
	Day 4	66.7%<br>204/306	-<br>**<br>ns<br>**	76.7%<br>253/330	**<br>-<br>ns<br>***	73.3%<br>222/303	ns<br>ns<br>-<br>***	47.2%<br>25/53	**<br>***<br>***<br>-
	Day 5	65.2%<br>204/313	-<br>ns<br>ns<br>***	62.4%<br>204/327	ns<br>-<br>ns<br>***	65.5%<br>203/310	ns<br>ns<br>-<br>***	26.8%<br>11/41	***<br>***<br>***<br>-
	Day 6	66.6%<br>207/311	-<br>**<br>*<br>*	77.8%<br>224/288	**<br>-<br>ns<br>***	75.4%<br>266/353	*<br>ns<br>-<br>***	50.0%<br>19/38	*<br>***<br>***<br>-
Monkey F	Day 1	59.8%<br>101/169	-<br>*<br>ns<br>ns	69.8%<br>127/182	*<br>-<br>ns<br>*	68.8%<br>128/186	ns<br>ns<br>-<br>*	50.0%<br>14/28	ns<br>*<br>*<br>-
	Day 2	58.9%<br>103/175	-<br>ns<br>ns<br>*	62.2%<br>122/196	ns<br>-<br>ns<br>*	61.7%<br>103/167	ns<br>ns<br>-<br>*	37.5%<br>9/24	*<br>*<br>*<br>-
	Day 3	56.5%<br>100/177	-<br>**<br>***<br>ns	71.3%<br>117/164	**<br>-<br>ns<br>ns	78.4%<br>149/190	***<br>ns<br>-<br>ns	70.8%<br>17/24	ns<br>ns<br>ns<br>-
	Day 4	70%<br>126/180	-<br>ns<br>ns<br>ns	69.2%<br>126/182	ns<br>-<br>ns<br>ns	71.9%<br>120/167	ns<br>ns<br>-<br>ns	60.7%<br>17/28	ns<br>ns<br>ns<br>-
	Day 5	74.9%<br>179/239	-<br>ns<br>ns<br>*	79.5%<br>178/224	ns<br>-<br>ns<br>**	77.4%<br>188/243	ns<br>ns<br>-<br>*	58.8%<br>20/34	*<br>**<br>*<br>-
	Day 6	67.8%<br>166/245	-<br>*<br>**<br>ns	77.0%<br>171/222	*<br>-<br>ns<br>ns	78.6%<br>187/238	**<br>ns<br>-<br>ns	65.7%<br>23/35	ns<br>ns<br>ns<br>-
	Day 7	65.7%<br>151/230	-<br>ns<br>ns<br>ns	66.4%<br>162/244	ns<br>-<br>ns<br>ns	71.9%<br>156/217	ns<br>ns<br>-<br>ns	69.8%<br>30/43	ns<br>ns<br>ns<br>-
	Day 8	59.2%<br>141/238	-<br>**<br>*<br>*	72.7%<br>168/231	**<br>-<br>ns<br>ns	69.3%<br>160/231	*<br>ns<br>-<br>ns	77.3%<br>34/44	*<br>ns<br>ns<br>-

…
(Continued on next page)
…

		Small Reward		Medium Reward		Large Reward		Jackpot Reward		Rare-Large / Common-Jackpot Reward	
		Successful/ Total Trials	Stats	Successful/ Total Trials	Stats	Successful/ Total Trials	Stats	Successful/ Total Trials	Stats	Successful/ Total Trials	Stats
Monkey E	Day 1	78.0%<br>177/227	-<br>*<br>ns<br>ns	87.0%<br>174/200	*<br>-<br>ns<br>**	81.9%<br>154/188	ns<br>ns<br>-<br>*	65.7%<br>23/35	ns<br>**<br>*<br>-		
	Day 2	74.9%<br>143/191	-<br>***<br>***<br>ns	87.7%<br>185/211	***<br>-<br>ns<br>*	89.7%<br>208/232	***<br>ns<br>-<br>**	69.2%<br>18/26	ns<br>*<br>**<br>-		
	Day 3	78.5%<br>150/191	-<br>**<br>***<br>ns	89.6%<br>189/211	**<br>-<br>ns<br>***	90.4%<br>206/228	***<br>ns<br>-<br>***	63.3%<br>19/30	ns<br>***<br>***<br>-		
	Day 4	77.6%<br>159/205	-<br>ns<br>*<br>ns	84.9%<br>185/218	ns<br>-<br>ns<br>ns	85.9%<br>176/205	*<br>ns<br>-<br>ns	70.8%<br>17/24	ns<br>ns<br>ns<br>-		
	Day 5	52.2%<br>142/272	-<br>***<br>***<br>ns	90.2%<br>305/338	***<br>-<br>ns<br>***	86.7%<br>241/278	***<br>ns<br>-<br>***	52.9%<br>18/34	ns<br>***<br>***<br>-		
	Day 6	51.0%<br>150/294	-<br>***<br>***<br>**	81.4%<br>232/285	***<br>-<br>ns<br>ns	84.6%<br>253/299	***<br>ns<br>-<br>*	73.1%<br>38/52	**<br>ns<br>*<br>-		
	Day 7	59.1%<br>104/176	-<br>***<br>***<br>ns	78.7%<br>159/202	***<br>-<br>ns<br>ns	81.7%<br>174/213	***<br>ns<br>-<br>ns	71.8%<br>28/39	ns<br>ns<br>ns<br>-		
	Day 8	47.4%<br>93/196	-<br>***<br>***<br>*	81.5%<br>172/211	***<br>-<br>ns<br>ns	80.6%<br>162/201	***<br>ns<br>-<br>ns	72.0%<br>18/25	*<br>ns<br>ns<br>-		
	Day 9	37.4%<br>52/139	-<br>***<br>***<br>*	77.6%<br>163/210	***<br>-<br>ns<br>ns	82.5%<br>170/206	***<br>ns<br>-<br>**	58.8%<br>20/34	*<br>ns<br>**<br>-		
	Day 10	49.8%<br>106/213	-<br>***<br>***<br>*	80.5%<br>186/231	***<br>-<br>ns<br>ns	80.8%<br>168/208	***<br>ns<br>-<br>ns	72.2%<br>26/36	*<br>ns<br>ns<br>-		
	Day 11	55.3%<br>94/170	-<br>***<br>***<br>ns	83.5%<br>177/212	***<br>-<br>ns<br>ns	85.8%<br>188/219	***<br>ns<br>-<br>*	71.9%<br>23/32	ns<br>ns<br>*<br>-		
Monkey E, standard reward range	All Days (9)	73.8%<br>1360/1844	-<br>ns<br>ns<br>***	75.9%<br>1421/1871	ns<br>-<br>ns<br>***	74.1%<br>1404/1894	ns<br>ns<br>-<br>***	48.8%<br>139/285	***<br>***<br>***<br>-		
Monkey F, precision task	All Days (29)	63.2%<br>2486/3933	-<br>***<br>***<br>ns	67.2%<br>2651/3946	***<br>-<br>***<br>ns	71.6%<br>2658/3711	***<br>***<br>-<br>***	63.3%<br>387/611	ns<br>ns<br>***<br>-		
Monkey F precision task, Mini-Jackpot sessions	All Days (28)	67.3%<br>1728/2569	-<br>***<br>***<br>***	78.9%<br>2030/2573	***<br>-<br>***<br>ns	83.3%<br>2092/2510	***<br>***<br>-<br>ns	80.6%<br>237/294	***<br>ns<br>ns<br>-		
Monkey E Rare-Large sessions	All Days (9)	54.5%<br>954/1752	-<br>***<br>***<br>***<br>***	83.7%<br>1610/1924	***<br>-<br>*<br>***<br>ns	86.4%<br>1397/1616	***<br>*<br>-<br>***<br>ns	72.1%<br>176/244	***<br>***<br>***<br>-<br>**	82.3%<br>214/260	***<br>ns<br>ns<br>**<br>-
Monkey E Common-Jackpot sessions	All Days (6)	52.1%<br>175/336	-<br>***<br>***<br>*<br>***	73.7%<br>261/353	***<br>-<br>*<br>ns<br>ns	80.9%<br>250/309	***<br>*<br>-<br>**<br>ns	66.7%<br>54/81	*<br>ns<br>**<br>-<br>ns	76.5%<br>289/378	***<br>ns<br>ns<br>ns<br>-

Table S3. Epoch success rate differences for Small to Large and Large to Jackpot rewards. Delay and Target Hold epochs correspond with Figure S5A and C (respectively), while reach epoch rates correspond with green bars in Figure 4B. Differences are shown on the top (black) with corresponding p-values (gray) below. Underlined values indicate significant differences ($p < 0.05$, binomial proportion test).

Change in epoch success rate
(% of trials that made it through the epoch)

**Epoch**	**Monkey N**		**Monkey F**		**Monkey E**	
	S to L	L to J	S to L	L to J	S to L	L to J
Delay	4.2	-4.1	0.79	-1.3	8.5	-10.8
	p < 1E-10	2E-5	0.22	0.29	< 1E-10	< 1E-10
Reach	4.5	-22.3	7.7	-10.9	18.3	-9.3
	1E-3	< 1E-10	7E-7	2E-4	< 1E-10	4E-9
Target Hold	1.6	-5.4	0.6	2.2	3.2	-0.2
	0.10	0.02	0.46	0.17	8E-7	0.89

Table S4. Specific failure mode rate differences for Small to Large and Large to Jackpot rewards. False Start and Delay Drift correspond with Figure S5A, Undershoot and Overshoot with Figure 4, and Hold Drift with Figure S5C. Differences are shown on the top (black) with corresponding p-values (gray) below. Underlined values indicate significant differences ($p < 0.05$, binomial proportion test).

Change in failure mode frequency
(% of trials that made it to the corresponding epoch)

**Failure Mode**	**Monkey N**		**Monkey F**		**Monkey E**	
	S to L	L to J	S to L	L to J	S to L	L to J
False Start	-2.2<br>p = 3E-7	2.9<br>1E-5	0.4<br>0.24	1.3<br>0.11	-6.4<br>< 1E-10	1.6<br>1E-3
Delay Drift	-2.0<br>2E-5	1.3<br>0.08	-1.2<br>0.02	-0.08<br>0.93	-2.1<br>8E-3	9.1<br>4E-9
Undershoot	5.3<br>1E-6	21.0<br>< 1E-10	-4.3<br>2E-3	11.6<br>2E-5	-7.1<br>< 1E-10	10.4<br>< 1E-10
Overshoot	-9.9<br>< 1E-10	1.3<br>0.44	-3.5<br>3E-5	-0.8<br>0.56	-11.2<br>< 1E-10	-1.1<br>0.33
Hold Drift	-1.6<br>0.1	5.4<br>0.02	-0.6<br>0.46	-2.2<br>0.17	-3.2<br>8E-7	-0.15<br>0.89

SI References

1. T. G. Lee, S. T. Grafton, Out of control: Diminished prefrontal activity coincides with impaired motor performance due to choking under pressure. *NeuroImage* 105, 145–155 (2015).
2. G. A. Rousselet, R. R. Wilcox, Reaction times and other skewed distributions: Problems with the mean and the median. *Meta-Psychology* 4 (2020).
3. A. P. Georgopoulos, J. F. Kalaska, J. T. Massey, Spatial trajectories and reaction times of aimed movements: effects of practice, uncertainty, and change in target location. *Journal of Neurophysiology* 46, 725–743 (1981).
4. T. Flash, N. Hogan, The coordination of arm movements: an experimentally confirmed mathematical model. *J. Neurosci.* **5**, 1688–1703 (1985).
5. C. L. MacKenzie, R. G. Marteniuk, C. Dugas, D. Liske, B. Eickmeier, Three-Dimensional Movement Trajectories in Fitts' Task: Implications for Control. *The Quarterly Journal of Experimental Psychology Section A* 39, 629–647 (1987).
6. D. Elliott, W. F. Helsen, R. Chua, A century later: Woodworth's (1899) two-component model of goal-directed aiming. *Psychological Bulletin* 127, 342–357 (2001).
7. R. S. Woodworth, Accuracy of voluntary movement. *The Psychological Review: Monograph Supplements* 3, i–114 (1899).
8. W. D. A. Beggs, C. I. Howarth, The accuracy of aiming at a target. *Acta Psychologica* 36, 171–177 (1972).
9. D. E. Meyer, S. Kornblum, R. A. Abrams, C. E. Wright, Optimality in human motor performance: Ideal control of rapid aimed movements. *Psychological Review* 95, 340–370 (1988).
10. D. Elliott, *et al.*, Goal-directed aiming: Two components but multiple processes. *Psychological Bulletin* 136, 1023–1044 (2010).
